# Tumor cell FAP orchestrates EMT and immune suppression in aggressive localized ccRCC

**DOI:** 10.7150/thno.118400

**Published:** 2026-01-01

**Authors:** Teijo Pellinen, Lassi Luomala, Kalle E Mattila, Annabrita Hemmes, Katja Välimäki, Mariliina Arjama, Oscar Brück, Lassi Paavolainen, Elisa Kankkunen, Harry Nisén, Petrus Järvinen, Leticia Castillon, Sakari Vanharanta, Paula Vainio, Olli Kallioniemi, Panu M. Jaakkola, Tuomas Mirtti

**Affiliations:** 1 Institute for Molecular Medicine Finland (FIMM), Helsinki Institute of Life Science (HiLIFE) and iCAN - Digital Precision Cancer Medicine Flagship, University of Helsinki, Helsinki, Finland.; 2 Department of Urology, Helsinki University Hospital and University of Helsinki, Helsinki, Finland.; 3 Department of Oncology and Radiotherapy, FICAN West Cancer Centre, University of Turku, Turku University Hospital, Turku, Finland.; 4 InFlames Research Flagship, University of Turku, Turku, Finland.; 5 Hematoscope Lab, Comprehensive Cancer Center & Center of Diagnostics, Helsinki University Hospital, Helsinki, Finland & Department of Oncology, University of Helsinki, Helsinki, Finland.; 6 Translational Cancer Medicine Program, Faculty of Medicine, University of Helsinki, Helsinki, Finland.; 7 Department of Biochemistry and Developmental Biology, Faculty of Medicine, University of Helsinki, Helsinki, Finland.; 8 Department of Pathology, Turku University Hospital, University of Turku, Turku, Finland.; 9 Science for Life Laboratory, Department of Oncology and Pathology, Karolinska Institute, Solna, Sweden.; 10 Department of Pathology, Diagnostic Center, Helsinki University Hospital, Helsinki, Finland.; 11 Research Program in Systems Oncology (ONCOSYS) and iCAN - Digital Precision Cancer Medicine Flagship, University of Helsinki, Helsinki, Finland.; 12 Finnish Cancer Institute, Helsinki, Finland.

**Keywords:** clear cell renal cell carcinoma, FAP, tumor microenvironment, epithelial-to-mesenchymal transition, spatial immunosuppression

## Abstract

**Background**: In contrast to most solid tumors, high immune cell infiltration in clear cell renal cell carcinoma (ccRCC) is associated with poor patient prognosis. The biological mechanisms underlying this paradox remain unclear, particularly regarding tumor cell-microenvironment interactions promoting local invasion and recurrence. This study aimed to identify spatially resolved tumor, immune, and stromal features that define aggressive phenotypes in localized ccRCC.

**Methods**: Multiplex immunofluorescence was performed using a 33-marker panel on 1,728 multi-region tissue cores from 435 surgically treated patients with localized ccRCC. Samples systematically included tumor centers, invasive borders, and adjacent benign tissue. Single-cell analyses quantified immune, stromal, endothelial, and epithelial cell populations within their spatial context.

**Results**: Spatially resolved profiling uncovered a highly aggressive tumor subtype distinguished by fibroblast activation protein (FAP) expression on tumor epithelial cells, a marker typically associated with stromal cells. Tumor-cell-specific FAP expression characterized an epithelial-to-mesenchymal transition (EMT)-like state and was spatially associated with profound immunosuppression, marked by enrichment of regulatory T cells, exhausted CD8+ T cells, and M2-like macrophages, particularly at the invasive border. Tumor-cell FAP promoted invasion and independently predicted significantly poorer recurrence-free survival (RFS), even in early-stage disease (multivariable Cox p = 0.022 for pT1-2), surpassing established biomarkers such as PD-L1 in capturing aggressive biological features.

**Conclusions**: Tumor epithelial FAP expression identifies an aggressive, immune-rich subtype of localized ccRCC, integrating EMT with spatially organized immunosuppression. These findings establish tumor-cell FAP as a promising biomarker with substantial translational potential for patient risk stratification, targeted imaging (FAPI-PET), and FAP-directed therapeutic strategies.

## Introduction

Localized clear cell renal cell carcinoma (ccRCC) carries significant recurrence risk despite surgery 1-3. Current therapies are limited by challenges in accurately selecting patients for intensified treatment or surveillance 4-6, highlighting an urgent need for robust prognostic biomarkers applicable across disease stages 7.

ccRCC tumors are highly vascularized and characterized by extensive immune infiltration 8. Paradoxically, while high angiogenesis signatures typically correlate with better survival, elevated immune infiltration often associates with poorer outcomes and therapy resistance, particularly in metastatic disease 8-14. Understanding this paradoxical association requires methods that can resolve complex spatial interactions between tumor cells and their microenvironment. Unlike conventional bulk analyses, spatially resolved techniques enable precise characterization of cell populations and their interactions at critical tumor regions, such as the invasive border, which may hold key insights into the mechanisms driving aggressive behavior and recurrence in localized ccRCC 15-17.

Recent studies suggest spatial interactions at tumor invasive margins involving cells undergoing epithelial-to-mesenchymal transition (EMT), immune cell subsets, and activated or myofibroblastic cancer-associated fibroblasts (CAFs) 17-19. These reports imply the existence of functionally specialized niches promoting local invasion and immune suppression. However, the detailed protein-level spatial organization and the precise cellular interplay responsible for driving disease recurrence remain poorly defined in localized ccRCC.

To specifically dissect the spatial tumor microenvironment (TME) characteristics linked with aggressive behavior in highly immune-infiltrated localized ccRCC, we employed multiplex immunofluorescence (mIF) on 1,728 multi-region tissue cores (tumor center, invasive border, adjacent benign) from 435 surgically treated patients. This spatially resolved analysis identified fibroblast activation protein (FAP) expression specifically on tumor epithelial cells as a defining feature of aggressive ccRCC. Although FAP has conventionally been viewed as a stromal marker, its unexpected expression by tumor cells was notably associated with hallmarks of EMT and a profoundly immunosuppressive niche. Importantly, tumor-specific FAP emerged as a robust independent predictor of disease recurrence in localized ccRCC.

## Materials and Methods

### Ethical approvals and data handling

This study adhered to the Declaration of Helsinki and was approved by the Institutional Review Boards of Helsinki University Hospital (Ethical Committee Helsinki University Hospital, diary number HUS/1040/2018) and Turku University Hospital (License number T06/032/15). Informed consent was waived per Finnish legislation allowing secondary use of anonymized health data.

### Patient cohorts and tissue microarrays

We utilized formalin-fixed, paraffin-embedded (FFPE) surgical specimens from 435 treatment-naïve patients with localized (N0M0) clear cell renal cell carcinoma (ccRCC) who underwent nephrectomy at Helsinki (n = 196) and Turku (n = 239) University Hospitals between 2003 and 2013. Exclusion criteria included distant (M1) or regional lymph node (N1) metastases, prior kidney cancer, or multiple synchronous kidney tumors. Specimens and corresponding tissue microarrays (TMAs) were previously collected and constructed 20. TMAs included replicate cores (1.0 mm Helsinki, 1.5 mm Turku) from tumor center (n = 2), invasive border (n = 2), and adjacent benign kidney tissue (n = 1). The detailed clinicopathological variables collected from medical records are summarized in Table S1.

### Multiplexed immunofluorescence (mIF)

We performed cyclic mIF using an established in-house protocol involving sequential rounds of antibody staining, imaging, and signal removal. Full antibody panel details and concentrations are in Table S2. The detailed step-by-step protocol with reagents is published in protocols.io (dx.doi.org/10.17504/protocols.io.rm7vz6775gx1/v1). Slides were scanned using Zeiss Axio Scan.Z1 with Colibri7 light source and filter set 112 (365 nm [DAPI], 488 nm, 555 nm, 647 nm, 750 nm).

### FAP antibody validation

FAP antibody specificity was validated using CRISPR-Cas9 knockout in the WPMY-1 myofibroblast cell line (ATCC CRL-2854). Lentiviral constructs 21 targeting *FAP* were produced and used to infect WPMY-1 cells. Following puromycin selection, *FAP* knockout was confirmed via immunohistochemistry.

### CRISPR-Cas9-mediated *FAP* silencing in HCC89 cells

Three single-guide RNAs (sgRNAs) targeting human *FAP* were cloned into a LentiCRISPRv2GFP plasmid (gift from David Feldser, Addgene plasmid # 82416) 22. The guides were the following: *FAP* A1 (exon 2) Forward: 5′-CACCGCAATAAGGCAAGCACAGCAG-3′, Reverse: 5′-aaacCTGCTGTGCTTGCCTTATTGC-3′; *FAP* A3 (exon 9) Forward: 5′-CACCGCTACAAAATATGCTCTCTGG-3′, Reverse: 5′ aaacCCAGAGAGCATATTTTGTAGC-3′; *FAP* A2 (exon 17) Forward: 5′-CACCGCCCATTTCCACCCTTCATGA-3′, Reverse: 5′-AAACTCATGAAGGGTGGAAATGGGC-3′. HCC89 cells were transduced with HelVi-BVC lentivirus (10 µl per well; 8 µg ml⁻¹ polybrene) and expanded in complete RPMI-1640 + 10% FBS. *FAP* knockout efficiency in A1/A2/A3 populations was assessed by anti-FAP-AF647 immunofluorescence.

### 3D collagen invasion assay

HCC89 parental and *FAP*-edited (A1/A2/A3) cells were seeded as 10,000 cells/well in 8-well chamber slides in 100 µL of 50% Matrigel (Corning, #356231, growth-factor-reduced) diluted in RPMI-1640 + 10% FBS. Spheroids were allowed to form for 72 h at 37 °C/5% CO₂. A 100 µl overlay of type-I collagen (1 mg/mL, Corning, #354236, rat tail) was then added on top; gels were allowed to polymerize for 1 h at 37 °C, followed by addition of 300 µl complete medium. Human TGF-β1 (PeproTech #200-01B) and Human IL-1β recombinant protein (PeproTech #100-21) were included at 1 ng/ml where indicated. Three independent invasion assays with different time point monitoring were performed (12 h, 24 h, 96 h). Phase-contrast imaging was performed at 10× magnification; images were calibrated at 0.667 µm/pixel (150 px = 100 µm). For analysis, images were processed in JupyterLab (Python): segmentation using either adaptive thresholding (Sauvola, parental/A3) or a ridge+edge fusion (A1/A2), skeleton pruning, and object labeling. Per-object geometric features were computed; the primary readout was perimeter (µm) per segmented invasive structure.

### Image processing and feature extraction

Scanned images (Zeiss*.czi*) were exported as TIFFs. Tissue cores were annotated and de-arrayed in FIJI/ImageJ. Cycle-to-cycle registration in MATLAB used the DAPI channel 23. Autofluorescent red blood cells were detected with ilastik (v1.3.3) 24 and excluded. Panel-specific ilastik classifiers generated epithelial (EpiMask) and stromal masks (TME panel: CA9/PanCK/E-cadherin for EpiMask; CAF panel 1: PDGFRB and αSMA for stroma). Nuclear segmentation on full-resolution DAPI employed the nucleAIzer deep-learning model 25. Nuclear masks were radially dilated by ~3 pixels within tissue masks to approximate whole-cell boundaries. Per-cell mean fluorescence intensities were then extracted for each channel using CellProfiler (v4.2.1) 26. Cell segmentation and phenotyping followed our published workflow 23, adapted to the present panels.

### Cell classification

All classifications were performed in Jupyter Notebook (Python 3.6.8). Marker positivity was determined using fixed intensity thresholds defined *a priori* by a pathologist (T.M.) and a multiplex-IF expert (T.P.) on representative tiles. Thresholds were reviewed against per-marker intensity histograms and raw grayscale overlays and locked before any outcome analyses. Final cell identities were assigned via rule-based combinations of marker positivity/negativity applied consistently across panels. Epithelial cells were defined as cells overlapping the ilastik-derived epithelial mask while not fulfilling criteria for leukocytes, endothelium, or stromal lineages. Stromal cells comprised non-epithelial, non-leukocyte, non-endothelial cells; when available (CAF panels), the ilastik-derived stroma mask supported this assignment. Leukocytes were defined as CD45-positive cells and further subclassified into T-cell subsets and myeloid populations using standard marker combinations (for example, CD45 with CD3 and CD8 or CD4 for T-cell lineages; FOXP3 for regulatory T cells; CD68 with CD163 for macrophages; and CD11c for dendritic-like myeloid cells). Endothelial cells were identified by CD31 positivity. Because fibroblast activation protein (FAP) is expressed in multiple compartments, FAP positivity was called separately within tumor-epithelial and stromal compartments using the same fixed thresholds. Throughout, we use “CD45⁺ leukocytes” to denote the pan-immune population and name immune subsets explicitly.

### Quantification of cell populations

Poor quality TMA cores were excluded per panel. Cell counts/proportions were averaged across replicate cores for each patient region. Proportions were calculated relative to relevant parent populations (e.g., % of Epi^-^ cells, % of CD45^+^ cells, % CD3^+^CD8^+^ cells) as indicated in figure/table legends. Cancer-associated fibroblasts (CAFs) were classified into 30 subsets (CAF1-30) based on combinatorial expression of PDGFRA, PDGFRB, FAP, αSMA (Panel 1) or other markers (Panel 2), adapting established systems 23, 27. Subset proportions were calculated relative to the total classified CAFs within the respective panel.

### Visual scoring of mesenchymal markers in tumor cells

Due to suboptimal automated detection for certain markers within epithelial regions, visual scoring was performed by two scientists (T.M., T.P., consensus) for specified mesenchymal markers (PDGFRA, PDGFRB, FAP, αSMA, SPARC, VIM, POSTN) and PD-L1 expression in tumor cells, guided by reference stains (H&E, PAX8, EpiStain). While PDGFRA/B, αSMA, and POSTN were negative, FAP, SPARC, VIM, and PD-L1 showed positivity in tumor cells. Expression in each tumor core (tumor center/border replicates) was scored as 0 (negative), 1 (weak), or 2 (strong). For specific analyses, these per-core scores were aggregated per patient using different methods (e.g., maximum score observed across cores, any positive score, or a cumulative score). The specific aggregation method used for each analysis is detailed in the relevant figure and table legends.

### Spatial heterogeneity testing (center vs. border)

We quantified intra-tumoral heterogeneity of tumor-cell FAP (ordinal 0/1/2) by comparing center and invasive border cores, restricting analyses to patients with both regions. We modeled FAP as a function of region with center as the reference, estimating the mean difference Δ(border-center) with patient-clustered robust standard errors (SEs). Models were fit separately within each cohort (Helsinki, Turku) and then in a pooled model that included a cohort indicator to account for baseline differences. We also tested a region×cohort interaction to evaluate whether the center-border difference varied between cohorts. Effects are reported as Δ with cluster-robust 95% CIs and Wald p-values.

### Duplicate-core reliability within regions

To assess sampling reliability independent of center-border differences, we compared duplicate cores within each region (center: c1 vs. c2; border: b1 vs. b2) using exact agreement, one-step agreement (|Δ| ≤ 1), and quadratic-weighted Cohen's κ. We also used paired Wilcoxon signed-rank tests to check for any systematic within-region shift.

### Statistical analyses

Statistical analyses were performed using GraphPad Prism (version 10.3.1), IBM SPSS Statistics (version 29.0.0.0), RStudio (version 2023.12.0) with R (version 4.2.2), and JupyterLab (version 3.0.16) with Python (version 3.6.15). In R, data manipulation and visualization used the tidyverse suite (version 2.0.0), including dplyr, ggplot2, readr (version 2.1.4), and readxl (version 1.4.3). The Mann-Whitney U test and Kruskal-Wallis test were used for non-parametric comparisons between two or more groups, respectively. Associations between categorical variables were assessed with Fisher's exact test or the chi-square test, as appropriate.

Region/interaction models were implemented in Python (statsmodels). For tumor-cell FAP heterogeneity (center vs. border), we used patient-clustered linear models with Huber-White (sandwich) standard errors to estimate the center-border difference and its 95% CI. For spatial immune-composition models, we fit linear mixed-effects models (statsmodels.formula.api.mixedlm) with a random intercept for patient and fixed effects for FAP (weak/strong vs. neg), region (border vs. center), core index (2 vs. 1), cohort, and the pre-specified FAP×region interaction for T cells. If a mixed model did not converge, we re-fit the identical specification by OLS with patient-clustered (Huber-White) SEs; in our data, the mixed model converged for exhausted CD8⁺, whereas Tregs and all myeloid features used the patient-clustered OLS fallback. When multiple comparisons were made, p values were adjusted using Bonferroni or controlled at 10% FDR with Benjamini-Hochberg.

For survival analyses. Kaplan-Meier (KM) curves and Cox proportional hazards (PH) models were performed in either R (survival/survminer) or Python (lifelines). The survival endpoint was from surgery to recurrence (RFS/MFS), and/or death (RFS), or end of follow-up. KM curves used log-rank tests. Multivariable Cox models were cohort-stratified when indicated (strata: Helsinki/Turku) and included tumor-cell FAP (weak vs. negative; strong vs. negative), age (binary cut at 65 years unless otherwise stated), pT (3-4 vs. 1-2), sex, grade, necrosis, and when stated continuous stromal features (CD31⁺, CD45⁺, stromal FAP%) entered as logits of their fractional areas. We used complete-case data, verified events-per-variable >10, assessed collinearity (all VIFs < 2), and checked PH with Schoenfeld residual tests (global and per-covariate). Sensitivity analyses when stated included the age cutoff (60/65/70 years) and treated pT as a covariate versus an additional stratum; results for FAP strong were directionally consistent.

## Results

### High immune infiltration correlates with poor prognosis in localized ccRCC

**Figure 1:**
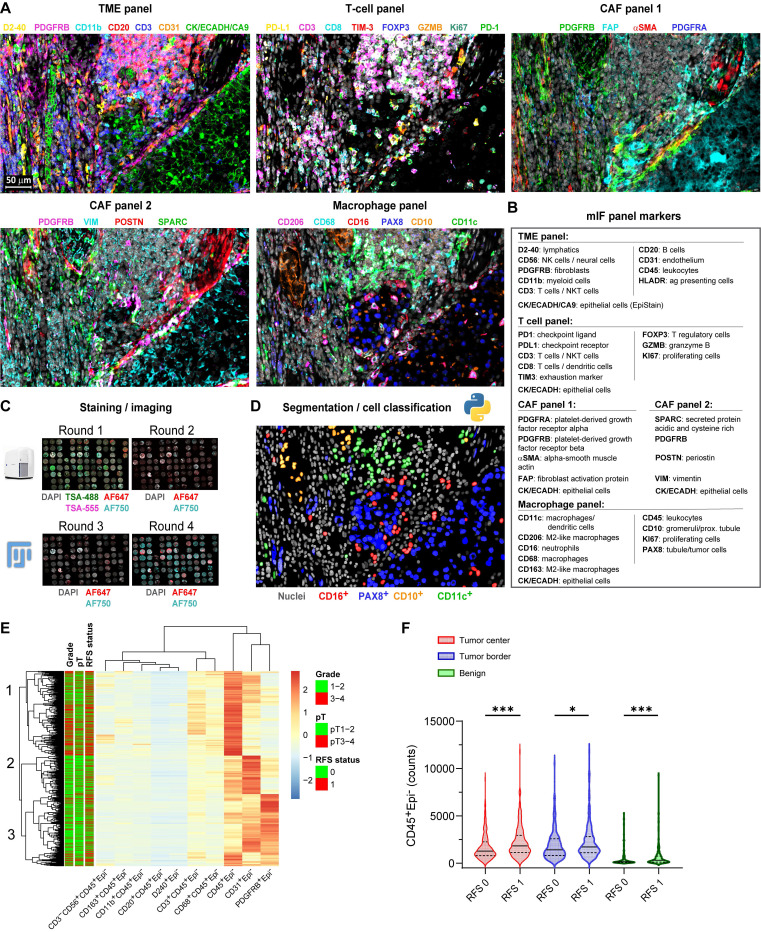
** Single-cell multiplex immunofluorescence reveals an immune-rich cell cluster associated with disease progression in localized ccRCC.** (**A**) Representative multiplex immunofluorescence (mIF) composite images from consecutive tissue sections of a ccRCC tumor border, stained with five antibody panels targeting immune and stromal markers. Scale bar represents 50 µm. (**B**) mIF antibody panels categorized by primary cellular targets for immune markers and by protein names for cancer-associated fibroblast (CAF) markers. (**C**) Overview of the multiplex IF protocol. Tyramide signal amplification (TSA) was used in the first staining and imaging cycle (channels AF488/AF555). Tissue microarray (TMA) cores were annotated, cropped using ImageJ/FIJI, and registered in MATLAB using DAPI as a spatial reference. (**D**) Example illustration of single-cell segmentation using nucleAIzer and subsequent cell classification based on mean intensity thresholding, exemplified with the macrophage panel. (**E**) Heatmap displaying unsupervised hierarchical clustering (Euclidean distance) of major immune (CD45⁺, CD3⁺, CD8⁺, CD20⁺, CD68⁺) and stromal (PDGFRB⁺, αSMA⁺, FAP⁺, CD31⁺) cell subset densities from individual TMA cores (total n = 1,728). Annotation bars indicate recurrence-free survival (RFS: 0 = non-recurrent; 1 = recurrent [metastasis or death]), pathological stage (pT1-2 vs. pT3-4), and nuclear grade (1-2 vs. 3-4). (**F**) Violin plots comparing CD45⁺Epi⁻ leukocyte cell counts between non-recurrent (RFS 0) and recurrent (RFS 1) patient cores from tumor center (n = 405), tumor border (n = 391), and adjacent benign areas (n = 355). Lines represent medians and interquartile ranges (IQR). ***p < 0.001, *p < 0.05 by two-sided Mann-Whitney U test. mIF = multiplex immunofluorescence; CAF = cancer-associated fibroblast; TSA = tyramide signal amplification; TMA = tissue microarray; AF = Alexa Fluor dye; RFS = recurrence-free survival; IQR = interquartile range.

We performed multiplex immunofluorescence (mIF) using 33 antibodies (Figure 1A-D) on 1,728 multi-region tissue cores from 435 localized ccRCC patients (Table S1), generating single-cell data on key TME populations. Unsupervised clustering based on immune and stromal cell densities identified three distinct tumor microenvironment (TME) subtypes characterized predominantly by CD45⁺ leukocytes, CD31⁺ endothelial cells, or PDGFRB⁺ stromal cells, respectively (Figure 1E). High CD45⁺ leukocyte cell density strongly associated with subsequent disease recurrence across all sampled regions (Mann-Whitney U test, p < 0.05; Figure 1F).

**Figure 2:**
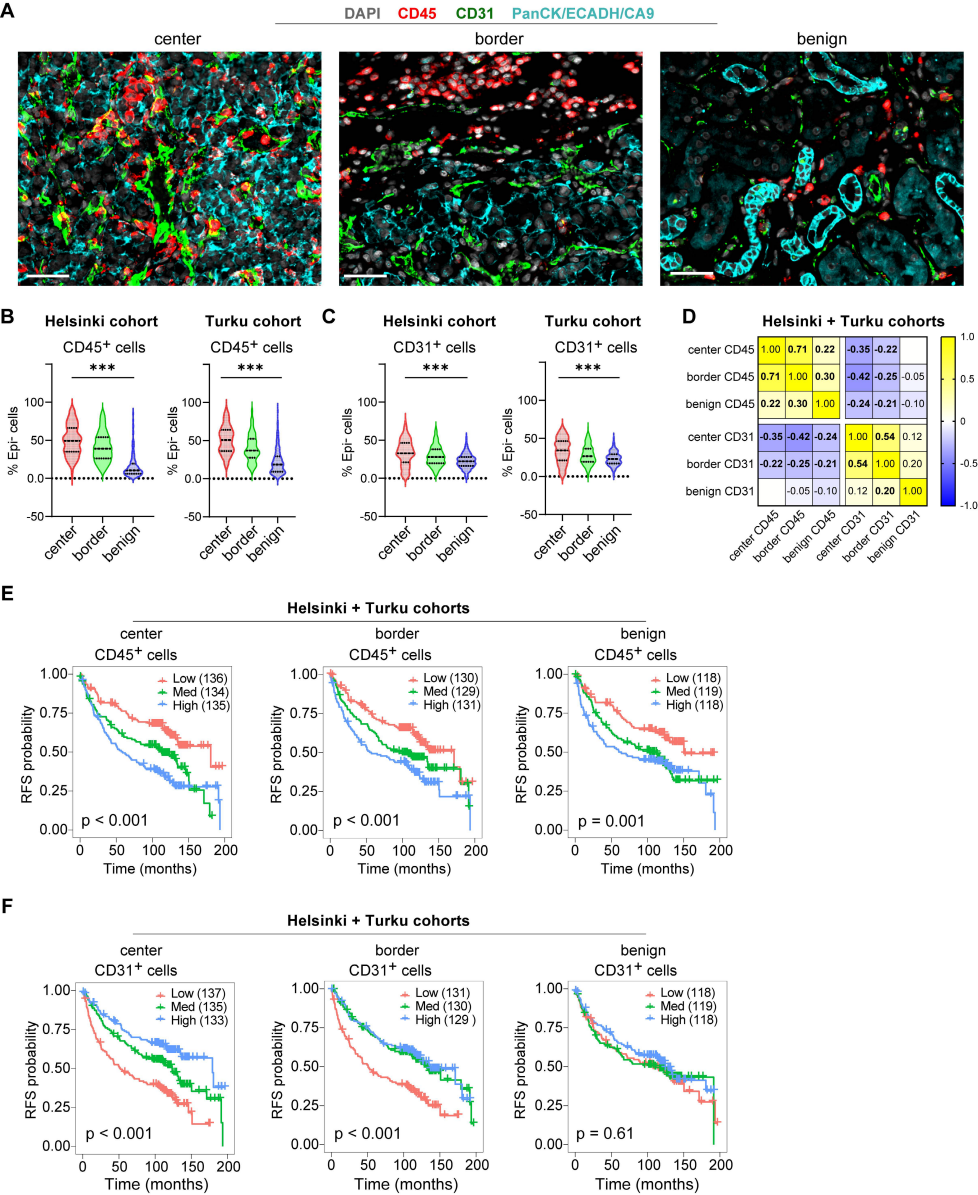
** Spatial distributions and prognostic associations of CD45⁺ and CD31⁺ cell densities in localized ccRCC.** (**A**) Example mIF images showing CD45 and CD31 staining alongside kidney epithelium stain (EpiStain) containing Pan-cytokeratin (PanCK), E-cadherin (ECADH), and carbonic anhydrase IX (CA9). Scale bar, 50 µm. (**B**) Relative densities of CD45^+^Epi⁻ cells as a proportion of total Epi⁻ cells in tumor center, border, and benign areas in Helsinki (n = 178) and Turku (n = 227) localized ccRCC cohorts. Boxes represent median and interquartile range; whiskers indicate 5th-95th percentiles. ***, p < 0.001 (Kruskal-Wallis test). (**C**) Similar to (B), but for CD31^+^Epi⁻ cells. (**D**) Correlation matrix (Pearson r) showing the relationship of CD45^+^Epi⁻ and CD31^+^Epi⁻ cell densities (as a proportion of total Epi⁻ cells) across tumor areas. Bold values indicate p < 0.05. (**E**) Kaplan-Meier curves for RFS stratified by tertiles of CD45⁺ density (as a proportion of total Epi⁻ cells) in tumor center, invasive border, and benign areas. Patient numbers per tertile are shown in parentheses. Log-rank p < 0.001 for all three regions. (**F**) Kaplan-Meier curves stratified by tertiles of CD31⁺ density (same cohort sizes and groupings), showing significant association with longer RFS in tumor center and border (Log-rank p ≤ 0.001) but not in benign regions (p = 0.61). RFS = recurrence-free survival.

Further characterization confirmed expected spatial distributions: Both leukocyte marking CD45⁺ cells and vascular endothelium marking CD31⁺ cells were more abundant in tumor regions compared to benign areas (Figure 2A-C), while these cell subsets showed a modest inverse correlation across all tissue samples (Figure 2D). Survival analyses on the merged cohort confirmed high CD45⁺ leukocyte infiltration predicted shorter RFS across all regions (Log-rank p ≤ 0.001; Figure 2E), while high CD31⁺ density predicted longer RFS, significantly only in tumor regions (Log-rank p < 0.001; Figure 2F). These opposing prognostic associations were validated by univariate Cox regression performed independently on the Helsinki and Turku cohorts (Figure S1A-C). Thus, high immune infiltration marks an aggressive TME subtype, prompting deeper investigation into tumor cell states and tumor microenvironment spatial interactions driving this poor prognosis in localized ccRCC.

### Tumor-specific FAP expression marks an EMT phenotype and stratifies immune-infiltrated ccRCC

**Figure 3:**
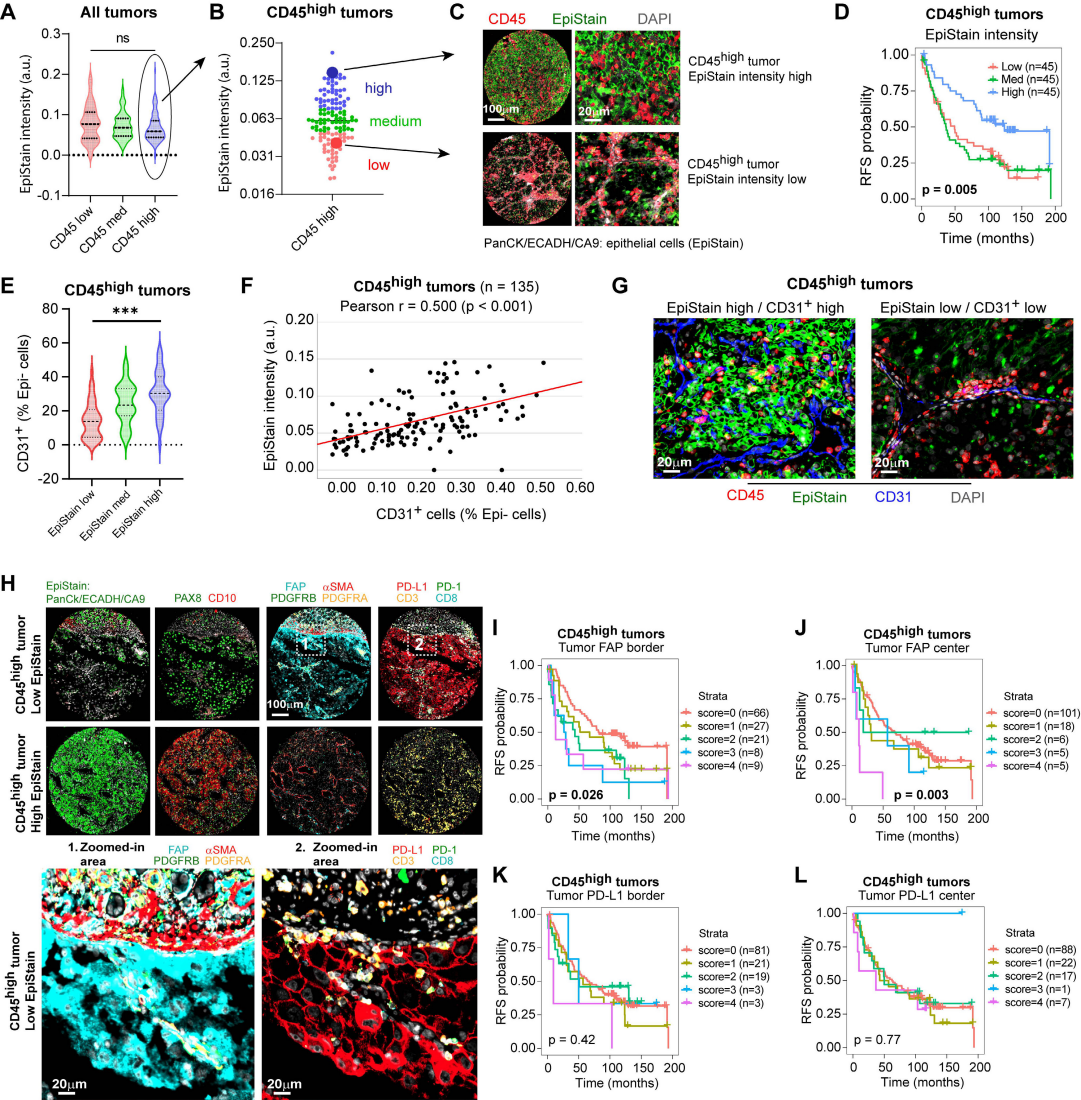
** Tumor-specific FAP expression marks an EMT phenotype and stratifies immune-infiltrated ccRCC.** (**A**) Distribution of pan-epithelial marker (EpiStain: CA9, E-cadherin, cytokeratins) intensity in tumor-center cores grouped by CD45⁺ density tertiles (low, med, high; each n = 135; total n = 405 tumors). Boxes denote median and interquartile range (IQR); whiskers indicate 5th-95th percentiles. p = 0.098 by two-sided Kruskal-Wallis test. (**B**) Classification of CD45^high^ tumors (n = 135) into EpiStain tertiles (low, med, high). (**C**) Representative mIF composite images of EpiStain (PanCK/E-cadherin/CA9) and CD45 in EpiStain^high^ versus EpiStain^low^ in CD45^high^ tumor centers. (**D**) Kaplan-Meier curves for recurrence-free survival (RFS) in CD45^high^ patients (n = 135), stratified by EpiStain tertiles. p = 0.005 by log-rank test. (**E**) Violin plots of CD31⁺ endothelial cell densities in CD45^high^ tumors (center) across EpiStain groups (n = 45 per group). Median and IQR are indicated; ***p < 0.001 by Kruskal-Wallis test. (**F**) Scatter plot of EpiStain intensity versus CD31⁺ density in CD45^high^ tumors (n = 135); Pearson r = 0.50, p < 0.001. (**G**) Representative mIF images of CD31⁺ endothelial cells and DAPI in EpiStain^low^ versus EpiStain^high^ CD45^high^ tumor centers. Scale bar, 20 µm. (**H**) mIF composites comparing CD45^high^ EpiStain^low^ (upper panel) and EpiStain^high^ (lower panel) phenotypes, highlighting tumor cell-specific FAP expression (Zoom 1) and PD-L1 expression (Zoom 2). Main panels scale bar, 100 µm; zooms, 20 µm. (**I, J**) Kaplan-Meier curves for RFS by cumulative tumor cell FAP score (0-4) in CD45^high^ tumors at invasive border (I; n = 131) and tumor center (J; n = 135). Patient numbers per score group are shown in parentheses. p = 0.026 (I) and p = 0.003 (J) by log-rank test. (**K, L**) Kaplan-Meier curves for RFS by tumor cell PD-L1 score (0-4) in CD45^high^ tumors at invasive border (K; n = 127) and tumor center (L; n = 135). No significant associations (p = 0.42 and p = 0.77, respectively) by log-rank test. mIF = multiplex immunofluorescence; ccRCC = clear cell renal cell carcinoma; EpiStain = pan-epithelial stain (CA9, E-cadherin, cytokeratins); RFS = recurrence-free survival; IQR = interquartile range.

While high leukocyte infiltration (CD45^high^) identifies a poor-prognosis group overall, these tumors exhibited considerable heterogeneity in epithelial marker expression (Figure 3A, B). We therefore investigated whether epithelial differentiation status could further stratify risk within this CD45^high^ cohort. Using a pan-epithelial marker cocktail (EpiStain: CA9, E-cadherin, cytokeratins), we categorized CD45^high^ tumors into low, medium, and high EpiStain groups based on expression in tumor centers (Figure 3A-C). Strikingly, this stratification revealed further survival differences within this cohort, with decreasing EpiStain predicting shorter RFS (overall Log-rank p = 0.005; Figure 3D). Further characterization revealed that EpiStain^low^ tumors possessed lower CD31⁺ densities (Figure 3E-G) as well as EMT-like features such as higher VIM (vimentin) and SPARC (secreted protein acidic and rich in cysteine) (Table 1). Notably, tumor-cell fibroblast activation protein (FAP) expression emerged as a key feature, significantly enriched in these highly immune infiltrated EpiStain^low^ cases, particularly at the tumor borders (p < 0.001; Table 1, Figure 3H).

To assess intra-tumoral spatial heterogeneity, we compared tumor-cell FAP (neg/low/high) between tumor center and invasive border. Within patients, borders showed higher FAP than centers (Δ(border-center) = 0.133; paired Wilcoxon p = 1.8×10⁻⁹, n = 411 patients; Figure S2A)). Category distributions corroborated this shift (more FAP-high cores at borders; 6.7% vs. 3.2%; Figure S2B). Duplicate cores within each region demonstrated high reliability (center: exact agreement 0.87, one-step 0.99, κ = 0.54; border: exact 0.81, one-step 0.97, κ = 0.56), and there was no systematic drift between duplicates (Wilcoxon p = 0.080 for centers; p = 0.738 for borders; Figure S2C). Cohort-specific, patient-clustered models confirmed consistent border enrichment (Helsinki Δ = 0.168, p = 3.8×10⁻⁶; Turku Δ = 0.090, p = 4.4×10⁻⁴) with a cohort-adjusted pooled effect Δ = 0.124 (p = 7.3×10⁻⁹), with no strong evidence that the border-center difference varied by cohort (interaction p = 0.077; Figure S2D). These analyses support border-enriched tumor FAP as a robust feature aligned with EMT-like, immune-rich tumor states.

Importantly, tumor FAP robustly predicted shorter RFS within the CD45^high^ group and the full cohort (Figure 3I-J; Figure S3A-B), while tumor PD-L1 showed no survival associations (Figure 3K-L; Figure S3C-D). Despite positive correlations with overall immune infiltration and tumor PD-L1 expression (Table S3, Figure 3H), tumor FAP offered additional prognostic stratification within the already poor-survival CD45^high^ patient group. This establishes tumor FAP as a key spatial biomarker intrinsically linked to highly aggressive, EMT-like phenotype of inflamed ccRCC.

### Tumor-cell FAP promotes 3D collagen invasion in ccRCC cells

**Figure 4:**
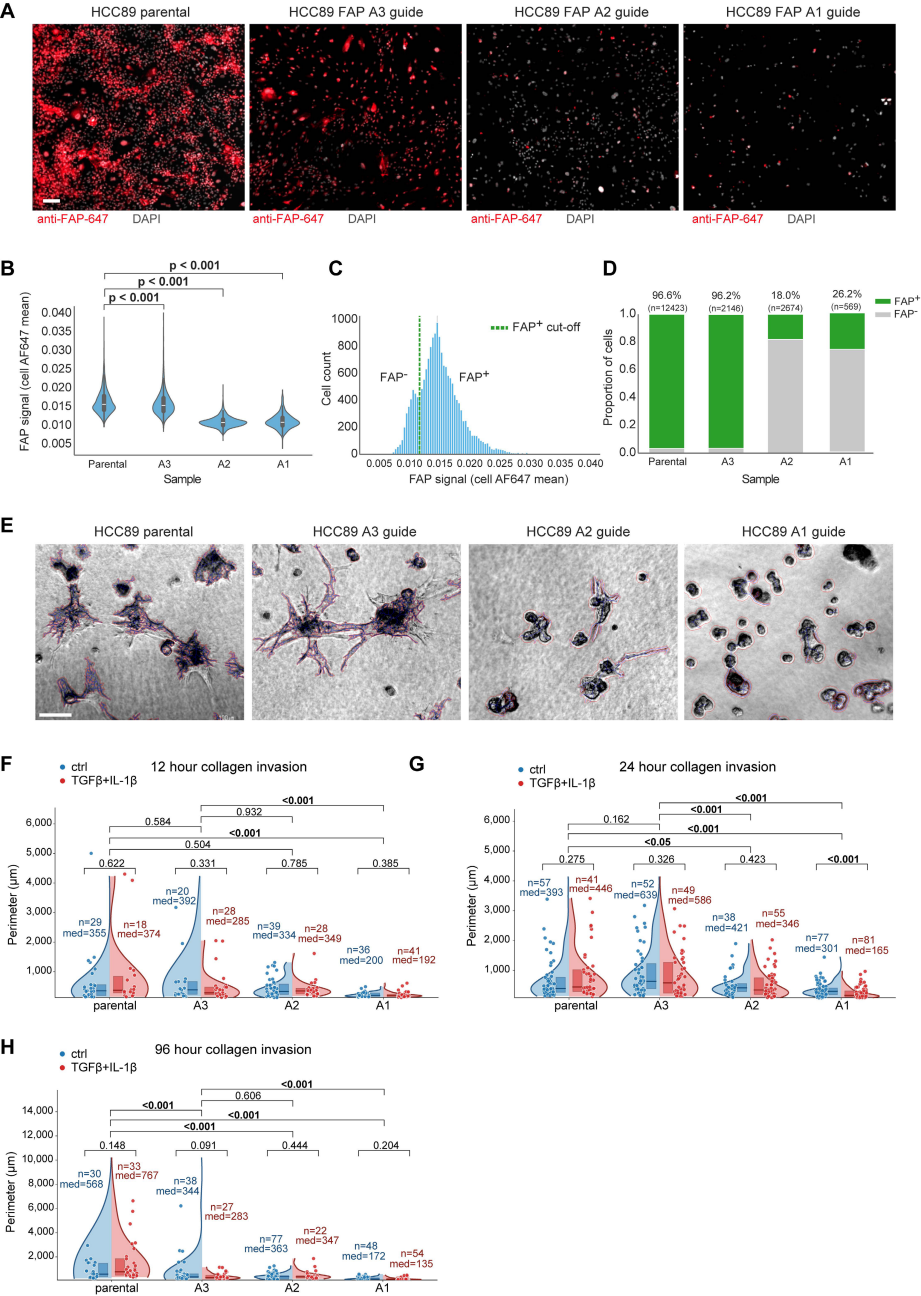
** Tumor-cell FAP promotes 3D collagen invasion in ccRCC cells.** (**A**) Immunofluorescence of HCC89 parental and CRISPR-Cas9 guides (FAP-A3, FAP-A2, FAP-A1). Anti-FAP-AF647 (red), DAPI (white). Scale bar, 50 µm. (**B**) Per-cell FAP intensity (AF647 mean) shown as violins with box overlays. P-values: pairwise two-sided Mann-Whitney U tests vs. parental with Benjamini-Hochberg FDR correction. (**C**) Pooled AF647 intensity histogram with the chosen FAP⁺ threshold (green dashed) determined by an automated KDE-valley method. (**D**) Per-image FAP⁺ fraction (mean ± bootstrap 95% CI); numbers above bars indicate cell counts (n). (**E**) 3D invasion assay phase-contrast examples (24 h time point). Cells were first allowed to form 3D spheroids for 48 h on Matrigel, then overlaid with type-I collagen (1 mg ml⁻¹) and imaged at 24 h. Parental/A3 form arborized protrusions; A1/A2 remain compact spheroids. Automated cellular component segmentation overlays (red) shown; see Methods. Scale bar, 100 µm. (**F**-**H**) Invasion quantification as split (half) violins per guide: ctrl (left, blue) vs. TGF-β+IL-1β (right, red, 1 ng ml⁻¹). Quartile boxes, median lines, and jittered field-level points are shown. The y-axis reports per-component perimeter (µm); “components” denote segmented connected cellular structures. Per-arm n values (number of components) and median perimeters are annotated. Two-sided Mann-Whitney U p-values.

To functionally test whether epithelial FAP drives an invasive phenotype, we edited *FAP*-positive HCC89 ccRCC cells (Figure S4A-B) with three independent CRISPR-Cas9 guides (FAP-A1/A2/A3) and compared them with parental cells in a 3D collagen-invasion assay. Cells were first allowed to form 3D spheroids on top of Matrigel (48 h), after which a collagen overlay (1 mg/ml) was applied. Efficient on-target editing was confirmed by immunofluorescence (IF) on 2D cultures: per-cell FAP intensity was markedly reduced in two guides (A1/A2), with a left-shift in the population intensity histogram and a decreased fraction of FAP-positive cells (Figure 4A-D). FAP antibody performance was validated also by loss-of-signal in IHC of a fibroblast cell line (Figure S4C).

Phase-contrast imaging in collagen revealed striking morphology differences: parental cells and the minimally edited A3 line formed arborized, stellate protrusions with long, branched networks, whereas *FAP*-KO lines (A1/A2) remained as compact, rounded spheroids with short or absent protrusions (Figure 4E). Quantitatively, in the 96-h time point the median invasive perimeter was 153.5 μm (A1) and 355 μm (A2) versus 667.5 μm in parental, corresponding to 23% (A1) and 53% (A2) compared with parental. These effects were consistent across fields and time points and remained significant under non-parametric testing with FDR control (Figure 4F-H). TGF-β + IL-1β (1 ng/ml each) did not systematically alter the invasion readout (Figure 4F-H). Together, these experiments provide causal functional evidence that epithelial FAP promotes a collagen-invasive phenotype in ccRCC cells.

### Coordinated tumor and stromal FAP expression defines an aggressive microenvironment

**Figure 5:**
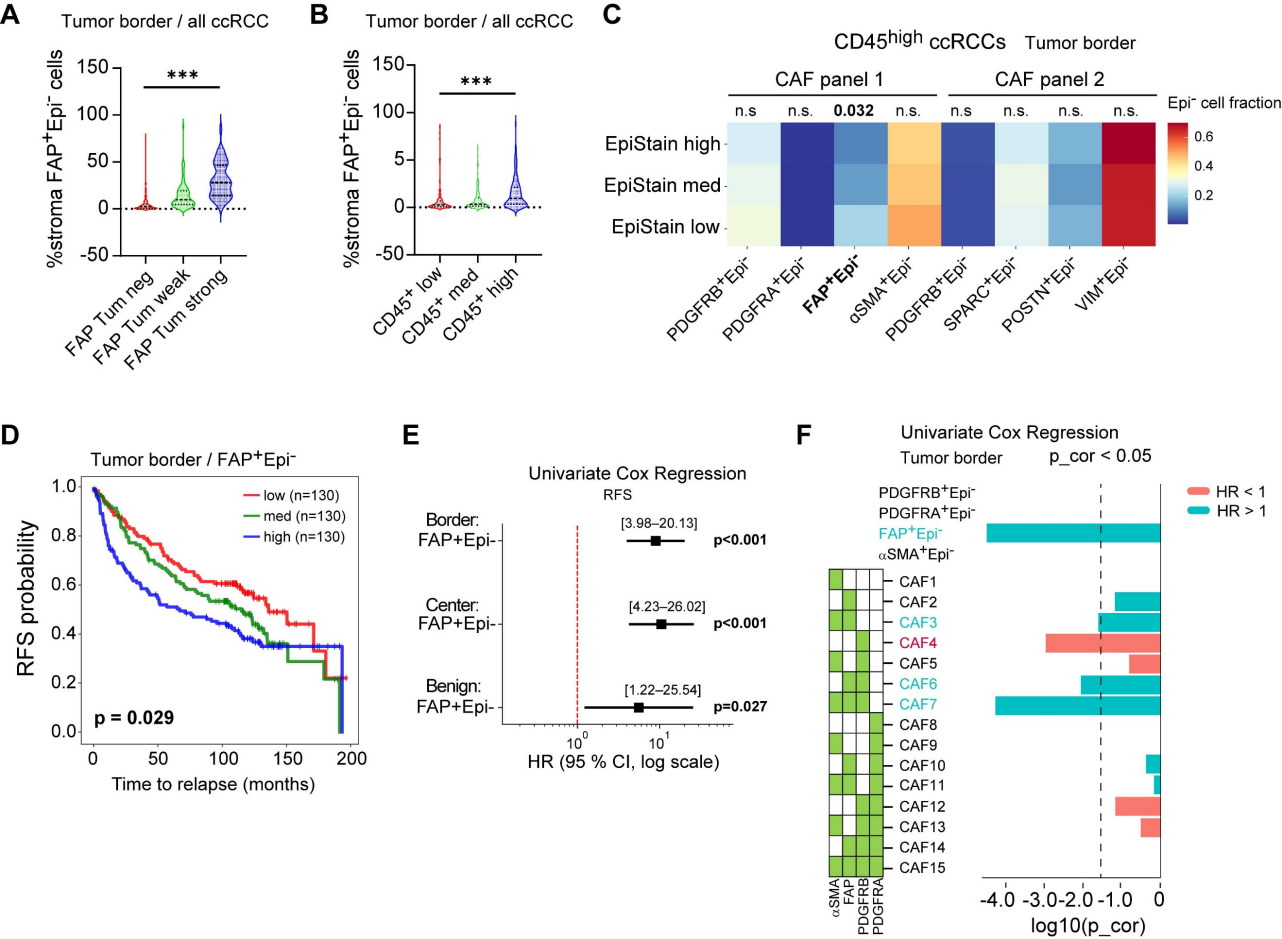
** Coordinated tumor and stromal FAP expression defines an aggressive microenvironment in localized ccRCC.** (**A**) Proportion of FAP⁺ stromal (Epi⁻) cells in tumor border cores stratified by tumor epithelial FAP expression level (negative, weak, strong; n = 394 ccRCC cases). Boxes denote median and interquartile range; whiskers indicate 5th-95th percentiles. ***P < 0.001 by two-sided Kruskal-Wallis test. (**B**) Proportion of FAP⁺ stromal cells in tumor border cores stratified by CD45⁺ density tertiles (low, med, high; n = 394). ***P < 0.001 by two-sided Kruskal-Wallis test. (**C**) Distribution of stromal subset proportions in tumor border cores of CD45^high^ ccRCCs (n = 135), across EpiStain tertiles (high, med, low). Bar plots show fraction of each subset (PDGFRB⁺, PDGFRA⁺, FAP⁺, αSMA⁺, SPARC⁺, POSTN⁺, VIM⁺) among total stromal (Epi⁻) cells. *p = 0.032 for FAP⁺ stromal cells by Kruskal-Wallis test; other comparisons not significant. (**D**) Kaplan-Meier curves for recurrence-free survival (RFS) stratified by tertiles of FAP⁺ stromal density in tumor border cores (n = 390; low, med, high: 130 each). p = 0.029 by log-rank test. (**E**) Forest plot of univariate Cox proportional hazards regression for continuous FAP⁺ stromal density predicting RFS in border (n = 390; HR [95% CI], log scale; p < 0.001), center (n = 404; p < 0.001), and benign (n = 353; p = 0.027) regions. (**F**) Forest plot of univariate Cox regression for proportions of indicated stromal subsets (all Epi⁻) in tumor border cores (n = 390). Subset names on the y-axis; log₂(HR) on the x-axis; green boxes indicate Bonferroni-corrected p < 0.05. CAF = cancer-associated fibroblast; Epi^-^ = non-epithelial; FAP = fibroblast activation protein; RFS = recurrence-free survival; HR = hazard ratio; CI = confidence interval; KW = Kruskal-Wallis; p_cor = Bonferroni-corrected p-value; EpiStain = pan-epithelial stain (CA9, E-cadherin, cytokeratins).

Investigating the interplay between FAP expression in different compartments, we observed that tumor-cell FAP positivity was closely associated with an increased abundance of FAP⁺ stromal cells, particularly at the invasive tumor border (Kruskal-Wallis test, p < 0.001; Figure 5A). FAP⁺ stromal cells were also enriched in CD45^high^ tumors (p < 0.001; Figure 5B). Within the CD45^high^ group, tumors with loss of epithelial marker expression (EpiStain^low^ cases) had higher FAP⁺ stromal cells (p = 0.032), an association not seen with other stromal markers (PDGFRA, POSTN, SPARC), indicating that stromal FAP specifically reflects an inflammation and EMT-associated response (Figure 5C). Increasing FAP⁺ stromal density associated with shorter RFS (overall Log-rank p = 0.029; Figure 5D), confirmed by Cox regression across tumor border and center (p < 0.001) as well as tumor adjacent benign regions (p = 0.027; Figure 5E). Further CAF subset spatial distribution analysis (Figure S5A-D) identified border-enriched myofibroblastic FAP⁺/αSMA⁺/PDGFRB⁺ subset (CAF7) predicting poor RFS (Cox p < 0.001; Figure 5F), while center-enriched single-marker POSTN⁺ cells predicted improved RFS (Cox p < 0.001, Figure S5E-H), highlighting spatial context importance for CAFs.

### Tumor FAP expression associates with a spatially organized immunosuppressive microenvironment

**Figure 6:**
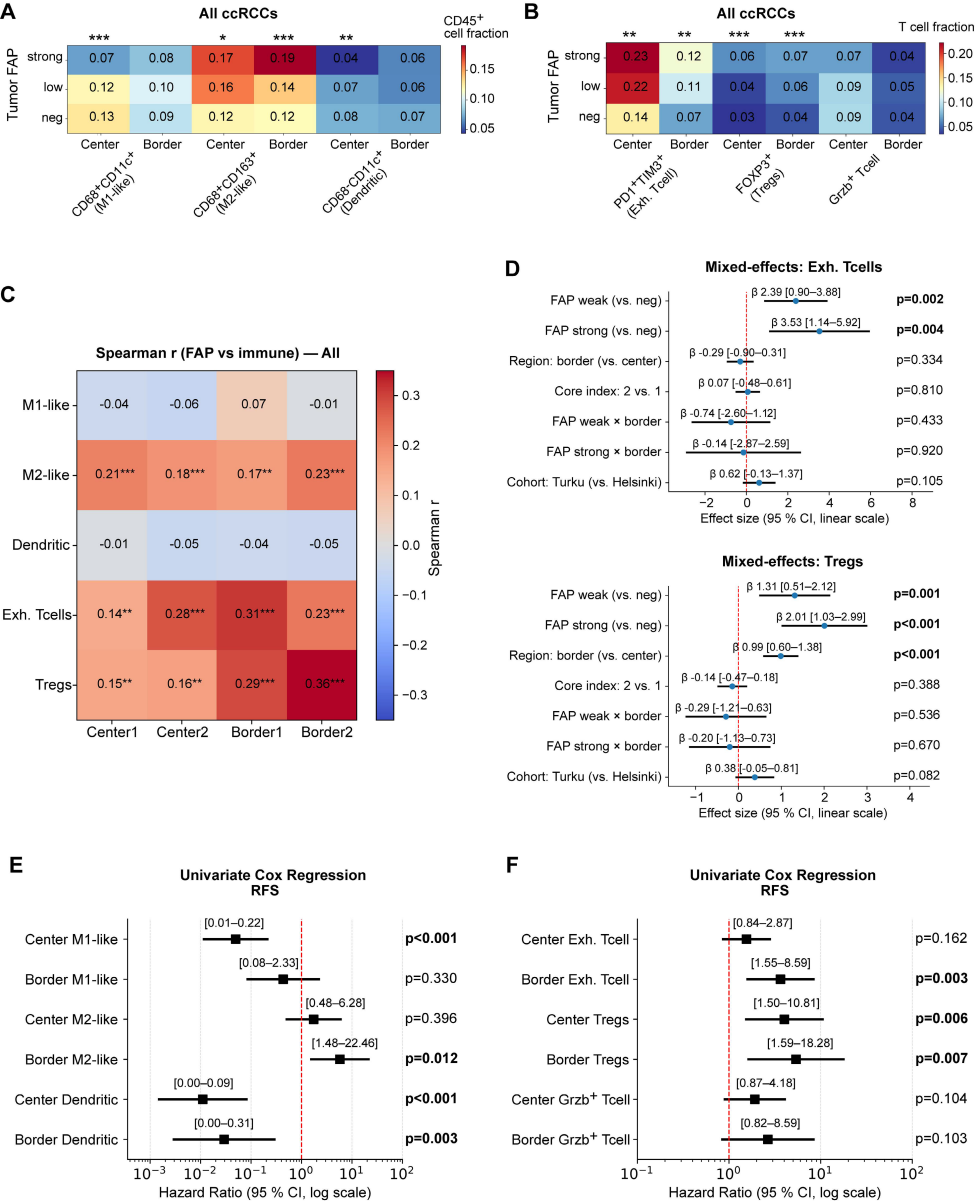
** Tumor-cell FAP relates to immune composition and spatial context.** (**A**) Heatmap of myeloid subset fractions (CD68⁻CD11c⁺, CD68⁺CD11c⁺, CD68⁺CD163⁺) among CD45⁺ cells, stratified by tumor-epithelial FAP score (neg/weak/strong). Averages shown separately for tumor center (n = 387 patients) and border (n = 374). Asterisks indicate two-sided Kruskal-Wallis across FAP groups: *p < 0.05, **p < 0.01, ***p < 0.001. (**B**) Heatmap of T-cell subset fractions (exhausted CD8⁺PD-1⁺TIM-3⁺, FOXP3⁺ Tregs, granzyme-B⁺ T cells) among CD3⁺ T cells, stratified by tumor FAP. Center: n = 397; border: n = 391. Significance as in (A). (**C**) Per-core Spearman correlations between tumor-cell FAP score and immune fractions across the four cores (Center1/Center2/Border1/Border2). Cells show Spearman *r*; asterisks denote FDR-adjusted significance (Benjamini-Hochberg) across the four cores within each feature (*q < 0.05, **0.01, ***0.001). (**D**) Linear mixed-effects models (random intercept for patient) for exhausted CD8⁺ T-cell fraction (upper) and Tregs (lower). Fixed effects: FAP (weak/strong vs. neg), region (border vs. center), core index (2 vs. 1), cohort, and FAP×region interaction. Points = β estimates; bars = 95% CI; p-values from Wald tests are shown at right. (**E**-**F**) Univariate Cox proportional hazards models for continuous immune fractions predicting RFS, shown separately for center and border. Left panel myeloid cells, right panel T cells. Points = HR; bars = 95% CI (log scale); p-values from Wald tests. Abbreviations: ccRCC, clear-cell renal cell carcinoma; FAP, fibroblast activation protein; RFS, recurrence-free survival; CI, confidence interval; HR, hazard ratio. *n* = patients; per-core analyses use all available cores.

We next examined how tumor FAP relates to immune composition and whether these associations depend on spatial context. Across all ccRCCs, higher tumor FAP scores were associated with a skewed myeloid landscape with more M2-like macrophages (CD163⁺) especially at the invasive border (Kruskal-Wallis p < 0.001) but fewer CD11c⁺ cells in the tumor center (p < 0.05; Figure 6A) and with increased fractions of terminally exhausted (PD-1⁺TIM-3⁺) CD8⁺ T cells and FOXP3⁺ regulatory T cells (Tregs) (p < 0.01; Figure 6B). These patterns persisted within CD45^high^ tumors (Figure S6A-B), indicating the effect is not driven by total leukocyte load.

At the per-core analysis, tumor-cell FAP correlated positively with Tregs and exhausted CD8⁺ T cells across all four cores (Center1/Center2/Border1/Border2) and with M2-like macrophages, while showing negative or null correlations with CD11c⁺ fractions (Figure 6C). Cohort-stratified correlation maps (Helsinki and Turku) showed the same pattern (Figure S6C-D).

To test whether these patterns reflect true spatial biology rather than between-patient differences, we fit multivariable linear mixed-effects models (LMM) with a patient-level random intercept, leveraging the four distinct TMA cores per patient. Fixed effects were tumor FAP category, region (center vs. border), TMA core index, and cohort (Helsinki/Turku), with prespecified FAP×region interactions for Tregs and exhausted CD8⁺ T cells, allowing us to estimate the independent effects of FAP and region while accounting for within-patient correlation. Exhausted CD8⁺ T cells and Tregs increased with FAP independent of cohort and region, and the region term was positive, confirming border-enrichment irrespective of FAP level (β = 1.31-3.53; joint wald test p ≤ 0.004; Figure 6D). Interaction terms (FAP×border) were small and non-significant (Wald
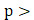
0.05), indicating that FAP association is present in both regions rather than being confined to the border. For myeloid subsets, FAP was region-independently associated with CD163+ M2-like macrophages (β = 0.49-0.95; Wald p ≤ 0.001; Figure S6E mid-panel), whereas M1-like cells showed lower values at the invasive borders (β = -0.24; p** =** 0.003; left panel). For CD11c+ dendritic cell fractions, the region effect did not reach significance in the LMM (Figure S6E, right-panel). Core-index effects were near zero, and duplicate-core reliability was moderate (ICC(2,1) = 0.3-0.56), supporting robustness of per-core estimates (Figure S6F-G).

Finally, univariate Cox models using continuous immune fractions showed that higher M2-like macrophages (Univariate Cox p = 0.012), exhausted T cells (Cox p = 0.003), and Tregs (Cox p = 0.007) predicted shorter RFS, especially when enriched at the tumor border, whereas higher center CD11c⁺ cells was associated with better outcomes (Cox p < 0.003) (Figure 6E-F). Collectively, these data link tumor-cell FAP to a spatially patterned immunosuppressive TME, characterized by higher Treg and exhausted CD8⁺ T cell fractions and a shift toward M2-like macrophages, with enrichment most pronounced at the invasive border.

### Tumor FAP independently predicts recurrence in ccRCC

**Figure 7:**
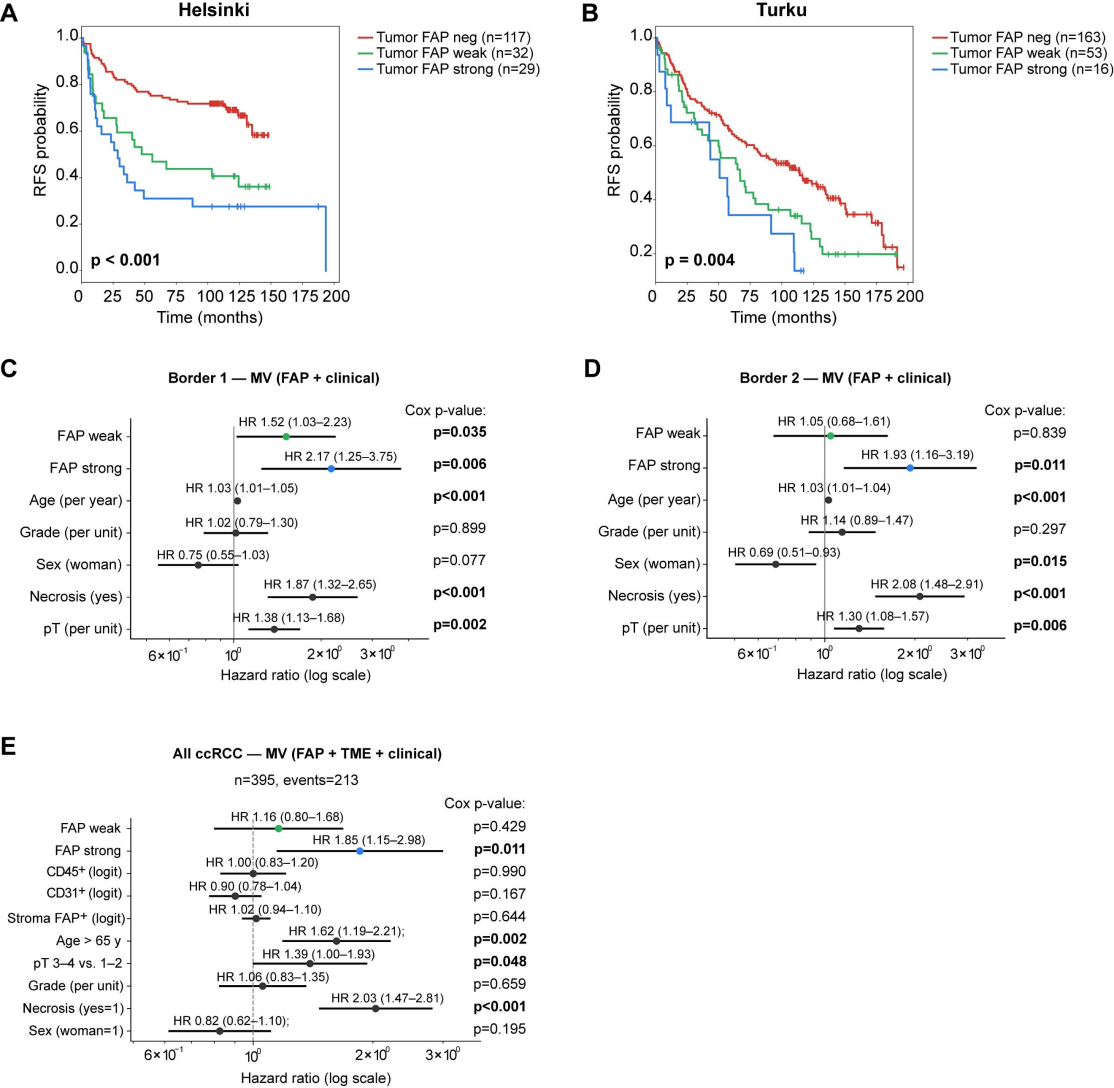
** Tumor FAP independently predicts recurrence in ccRCC.** (**A**-**B**) Kaplan-Meier curves for recurrence-free survival (RFS) by three-tier tumor-cell FAP score (negative, weak, strong) in the Helsinki (A) and Turku (B) cohorts (max value across cores per patient). Log-rank p-values shown. (**C**-**D**) Multivariable Cox models for RFS in tumor-border core sets 1 (C) and 2 (D). Models are stratified by cohort (Helsinki/Turku) and adjust for pT (ordinal, 1-4), necrosis (yes/no), sex (woman = 1), and grade (ordinal, 1-4). HRs (95% CIs) are printed above each line; p-values are shown at right. (**E**) Cohort-stratified *joint* Cox model combining both cohorts and adjusting for clinical covariates and stromal features. Covariates: tumor FAP (weak vs. negative; strong vs. negative), age > 65 y (vs. ≤ 65 y), pT 3-4 (vs. 1-2), necrosis (yes/no), sex (woman = 1), grade (ordinal), and stromal metrics entered as continuous logit-scaled fractions: CD31⁺ area, CD45⁺ area, and stromal FAP%. HRs (95% CIs) are shown above lines with Cox Wald p-values at right. The model satisfied proportional-hazards assumptions (global Schoenfeld p = 0.09) and had adequate events-per-variable (n = 21). n = 395 (events = 213). Abbreviations: FAP, fibroblast activation protein; RFS, recurrence-free survival; HR, hazard ratio; CI, confidence interval; neg, negative; MV, multivariable.

We next evaluated the clinical relevance of tumor FAP expression in ccRCC. In Kaplan-Meier analyses with three-tier categorisation, positive tumor FAP was associated with shorter RFS both in Helsinki (n = 178) and Turku (n = 222) cohorts separately (log-rank, p ≤ 0.004 for both; Figure 7A-B). The association was also observed when tumor FAP was analyzed separately in spatially distinct tumor-border cores within each cohort (log-rank, p < 0.001 and p = 0.046; Figure S7A-B). To spatially and clinically validate these findings, we performed multivariable Cox regression in the two tumor-border foci of the same cases: strong tumor FAP remained independently associated with shorter RFS in both foci (Cox p = 0.006 and 0.011; Figure 7C-D).

To evaluate independence from clinical and stromal features, we constructed a cohort-stratified multivariable Cox model including age, sex, pT, grade, necrosis, and continuous logit-scaled stromal metrics (CD31⁺, CD45⁺, stromal FAP%). In this joint model, strong tumor-cell FAP remained independently associated with shorter RFS (Figure 7E), with consistent effect sizes across alternative age cut-points and pT handling (Figure S7C).

We then focused on early-stage disease (pT1-2; n = 302). Strong tumor FAP showed strong univariate association with shorter RFS (Cox p < 0.001; Figure S8A) and remained significant in Kaplan-Meier analyses performed on the combined cohort (log-rank p < 0.001; Figure S8B) and within each cohort separately (log-rank p ≤ 0.031; Figure S8C-D). Crucially, strong tumor FAP remained an independent predictor of RFS in multivariable regression (Cox p = 0.022; Figure S8E). The effect was even stronger for metastasis-free survival (MFS) (Cox p < 0.001; Figure S8F), where tumor FAP outperformed age as a predictor of metastatic potential. Collectively, tumor-cell FAP is a robust, independent prognostic marker in ccRCC, including early-stage disease.

## Discussion

This study leveraged multi-region, single-cell spatial profiling to dissect the complex tumor microenvironment of localized clear cell renal cell carcinoma (ccRCC), revealing an unexpected and central role for fibroblast activation protein (FAP) expressed directly on tumor epithelial cells. While conventionally viewed as a stromal marker, our findings establish that tumor-cell FAP expression identifies a distinct and aggressive subset of localized ccRCC. This phenotype, particularly enriched in highly immune-infiltrated tumors, integrates key hallmarks of poor prognosis: an epithelial-to-mesenchymal transition (EMT)-like state, profound immune suppression characterized by M2-like macrophage and regulatory T cell enrichment, T cell exhaustion, and reduced angiogenesis. Crucially, tumor FAP emerged as a robust independent predictor of recurrence-free survival, even in early-stage disease, highlighting its potential role in early tumor progression.

Our analysis confirmed key TME prognostic associations (high CD45⁺ correlating with shorter RFS and high CD31⁺ with longer RFS) within localized ccRCC, extending observations previously made largely in metastatic settings 8-14. However, we moved beyond this by showing tumor FAP expression effectively stratifies the prognostically heterogeneous CD45^high^ group. Tumor-cell FAP integrates multiple converging adverse processes (EMT, immune evasion including PD-L1 co-expression, impaired angiogenesis), encapsulating the core biology of this aggressive immune-rich phenotype in a single spatial marker.

Tumor FAP association with low EpiStain and other mesenchymal markers provides strong, spatially resolved protein-level evidence for an EMT-like program in aggressive localized ccRCC. FAP marked this state more robustly than traditional EMT markers prognostically, perhaps reflecting a distinct EMT program linked to immune modulation 17, 19. Frequent PD-L1 co-expression further suggests tumor FAP signifies cells undergoing EMT while engaging adaptive immune resistance. Our EMT findings in ccRCC progression build on earlier transcriptomic studies 12, 14 by demonstrating protein-level changes in localized tumors. Consistent with an invasion-competent EMT state, CRISPR-Cas9 *FAP* knockout in HCC89 spheroids attenuated 3D collagen I invasion, supporting a cell-autonomous role for tumor-cell FAP in invasion.

We explicitly assessed intra-tumor spatial heterogeneity and found higher tumor-cell FAP at the invasive border relative to tumor centers, with consistent effects across cohorts. This inter-regional pattern aligns with the observed enrichment of immunosuppressive niches at the border. Intriguingly, tumor-cell FAP expression strongly correlated with the abundance of FAP⁺ CAFs, with spatial colocalization particularly evident at the invasive border. This co-occurrence, alongside the co-enrichment of both FAP⁺ tumor cells and FAP⁺ CAFs in immune-rich (CD45^high^) and EMT^high^ (EpiStain^low^) tumors, suggests a shared induction mechanism - a “field effect” in which immune-driven inflammation may up-regulate FAP via cytokines such as IL-1β, TGF-β, or CCL2 28-31. However, our detailed CAF subtyping also revealed significant spatial and functional heterogeneity within the stroma. While myofibroblastic FAP⁺/αSMA⁺ CAFs (e.g., CAF7) enriched at the border correlated strongly with poor prognosis, echoing findings in other cancers 19, 23, 27, POSTN⁺ CAFs specifically within the tumor center were linked to improved survival, potentially aligning with reports of antigen-presenting CAF functions 19. This highlights the importance of CAF subtype and spatial location when assessing stromal roles in ccRCC progression.

The microenvironment associated with high tumor-cell FAP expression was profoundly immunosuppressive, characterized by skewed myeloid populations (increased M2-like CD68⁺CD163⁺ macrophages, decreased CD11c⁺ myeloid cells) and accumulations of FOXP3⁺ regulatory T cells (Tregs) and terminally exhausted (PD-1⁺TIM-3⁺) CD8⁺ T cells. Importantly, these immunosuppressive elements were specifically enriched at the invasive tumor border regions in tumors with strong tumor-cell FAP expression. Survival analyses underscored the prognostic significance of this spatial organization: higher densities of M2-like macrophages, exhausted CD8⁺ T cells, and Tregs at the tumor border predicted significantly shorter recurrence-free survival. Conversely, tumors with low FAP expression in the tumor center exhibited enrichment of CD11c⁺ myeloid cells, correlating with improved outcomes. Thus, tumor-cell FAP expression is not only indicative of immune dysfunction but spatially coordinates this dysfunction specifically at the invasive front, suggesting immune evasion mechanisms concentrated at critical tumor-host interaction sites. Remarkably, these associations persisted even within the CD45^high^ subgroup, emphasizing that tumor-cell FAP captures distinct aspects of immune dysfunction independent of overall immune infiltration levels.

Our findings position tumor FAP as a compelling biomarker. Its independent prediction of recurrence, particularly in early-stage ccRCC, suggests utility for risk stratification and potentially guiding adjuvant therapy selection. Non-invasive FAPI-PET imaging 32 could enable real-time risk assessment. Furthermore, FAP is an attractive therapeutic target 33-35, and our data showing its dual expression on both aggressive tumor cells and 'activated' CAFs provide strong rationale for investigating FAP-targeted therapies in this high-risk subtype. In addition, consistent with an invasion-competent EMT state, CRISPR-Cas9 knockout of *FAP* in ccRCC spheroids reduced 3D collagen invasion, providing causal, cell-autonomous support for tumor-cell FAP as a driver of invasive behavior and aligning with our tissue-level EMT/immunosuppression phenotype.

The limitation of this study includes the reliance on multi-region TMAs, which inherently sample a limited field of view. To mitigate this, we systematically analyzed two center and two border cores per patient, demonstrated high within-region reliability and consistent border-center differences across two independent cohorts, and replicated prognostic associations in duplicate border cores as well as in cohort-stratified multivariable models. Spatial mixed-effects analyses and orthogonal functional invasion assays further support the biological conclusions. Nevertheless, prospective validation on whole-slide sections and in biopsy cohorts will be important to confirm generalizability and to facilitate clinical implementation. Because FAP and immune subsets were measured on different sections, we could not compute single-cell proximity/neighbor statistics between FAP⁺ tumor cells and immune cells; our region- and core-level analyses with mixed-effects modeling partially address spatial relationships. Future work will implement same-section multiplexing to enable per-cell nearest-neighbor/co-occurrence tests. In addition, functional validation was limited to an in vitro 3D spheroid invasion assay in a single ccRCC line that does not model immune crosstalk and additional in vivo studies will be needed to define mechanism and generalizability. Mechanisms driving tumor FAP expression warrant further study.

## Conclusions

Our spatial analysis identifies tumor epithelial FAP expression as a hallmark of aggressive, highly immune-infiltrated localized ccRCC. Tumor FAP integrates EMT programs with a spatially organized immunosuppressive TME and independently predicts recurrence. These findings highlight spatial context importance and position tumor FAP as a promising biomarker and therapeutic target. Future research should utilize these insights to guide biomarker-driven therapeutic decisions for localized ccRCC.

## Supplementary Material

Supplementary figures and tables.

## Figures and Tables

**Table 1:** Association between epithelial differentiation (EpiStain) and mesenchymal marker tumor cell expression in CD45^high^ ccRCC (n = 135).

Mesenchymal marker (tumor border)	EpiStain low	EpiStain med	EpiStain high	p-value
**FAP** (tumor)				< 0.001
Negative	13 (28%)	31 (61%)	23 (68%)	
Positive	34 (72%)	20 (39%)	11 (32%)	
**SPARC** (tumor)				0.001
Negative	16 (36%)	36 (71%)	22 (67%)	
Positive	29 (64%)	15 (29%)	11 (33%)	
**VIM** (tumor)				0.018
Negative	2 (4%)	13 (25%)	6 (18%)	
Positive	43 (96%)	38 (75%)	27 (82%)	
**PD-L1** (tumor)				0.002
Negative	20 (46%)	33 (65%)	28 (85%)	
Positive	23 (54%)	18 (35%)	5 (15%)	

^a^EpiStain intensity was quantified in tumor-center cores (2 replicates) for CD45^high^ patients (n = 135) and classified into equal tertiles (low/med/high) by mean intensity.^b^Mesenchymal markers were scored in tumor-border cores (2 replicates); any tumor-cell positivity in either core was recorded as positive.^c^Cases with missing data were excluded (FAP: n = 3; SPARC: n = 6; VIM: n = 6; PD-L1: n = 8).p-values by two-sided Pearson chi-square test.

## Abbreviations

CAF: cancer-associated fibroblast
CA9: carbonic anhydrase IX
ccRCC: clear cell renal cell carcinoma
CI: confidence interval
DAPI: 4′,6-diamidino-2-phenylindole
ECADH: E-cadherin
EMT: epithelial-to-mesenchymal transition
EpiStain: pan-epithelial stain (CA9, E-cadherin, cytokeratins)
FAP: fibroblast activation protein
FAPI-PET: fibroblast activation protein inhibitor positron emission tomography
FDR: false discovery rate
FFPE: formalin-fixed paraffin-embedded
H&E: haematoxylin and eosin
HR: hazard ratio
ICC: intraclass correlation coefficient
IHC: immunohistochemistry
IF: immunofluorescence
IL-1β: interleukin-1 beta
IQR: interquartile range
KM: Kaplan-Meier
KO: knockout
LMM: linear mixed-effects model
mIF: multiplex immunofluorescence
MFS: metastasis-free survival
MV: multivariable
OLS: ordinary least squares
PD-1: programmed cell death protein 1
PD-L1: programmed death-ligand 1
PH: proportional hazards
RCC: renal cell carcinoma
RFS: recurrence-free survival
TGF-β: transforming growth factor beta
TMA: tissue microarray
TME: tumor microenvironment
Tregs: regulatory T cells
TSA: tyramide signal amplification
