# Supplementary material for: Tumor cell FAP orchestrates EMT and immune suppression in aggressive localized ccRCC
Source: Theranostics. 2026 Jan 1;16(7):3246–62. doi: 10.7150/thno.118400 (PMC12846009; doi:10.7150/thno.118400)
Supplement: Supplementary file 1 — Supplementary figures and tables. [file thnov16p3246s1.pdf]

Supplementary Material

Tumor cell FAP orchestrates EMT and immune suppression in aggressive localized ccRCC

AUTHORS

Teijo Pellinen[1,*], Lassi Luomala[2], Kalle E Mattila[3,4], Annabrita Hemmes[1], Katja Välimäki[1], Mariliina Arjama[1], Oscar Brück[5], Lassi Paavolainen[1], Elisa Kankkunen[2], Harry Nisén[2], Petrus Järvinen[2], Leticia Castillon[6], Sakari Vanharanta[6,7], Paula Vainio[8], Olli Kallioniemi[1,9], Panu M. Jaakkola[3], Tuomas Mirtti[10,11]

Table S1: Clinicopathological characteristics of patients

Table S2: Composition and staining conditions of five multiplex immunofluorescence antibody panels

Table S3: Association of tumor epithelial FAP expression with epithelial marker intensity, immune and endothelial cell densities, and PD-L1 in localized ccRCC (n = 418)

Figure S1: Univariate Cox regression of stromal and immune subset densities predicting recurrence-free survival in ccRCC

Figure S2: Spatial heterogeneity and within-region reliability of tumor-cell FAP

Figure S3: Tumor cell FAP but not PD-L1 expression stratify recurrence-free survival in localized ccRCC

Figure S4: Validation of FAP antibody specificity and FAP expression profiling in cancer cell lines.

Figure S5: Distribution and prognostic associations of multi-marker defined CAF subsets in localized ccRCC

Figure S6: Cohort-specific correlations, mixed-effects for myeloid, and duplicate-core reliability

Figure S7: Supplemental survival analyses for tumor-cell FAP

Figure S8: Tumor FAP independently predicts recurrence in early-stage ccRCC

Table S1: Clinicopathological characteristics of patients.

Variable	Helsinki (n = 196)	Turku (n = 239)
**Median follow-up time, years**[a] (IQR)	8.69 (2.08–10.23)	6.01 (2.09–9.86)
**5-year RFS rate**	62%	62%
**5-year OS rate**	81%	73%
**Age at diagnosis**, mean (years)	64	67
**Sex**		
Male	99 (51%)	142 (59%)
Female	97 (49%)	97 (41%)
**Pathological stage (pTNM)**		
pT1a-b	87 (44%)	136 (57%)
pT2a-b	26 (13%)	53 (22%)
pT3-T4	83 (42%)	50 (21%)
**Grade**[b]		
1	14 (7%)	14 (6%)
2	105 (54%)	118 (49%)
3	67 (34%)	86 (36%)
4	10 (5%)	15 (6%)
Not available (na)	–	6 (3%)
**Tumor necrosis**		
No	132 (67%)	135 (56%)
Yes	58 (30%)	103 (43%)
Not available (na)	6 (3%)	1 (0%)

Abbreviations: RFS, recurrence-free survival; OS, overall survival; IQR, interquartile range; na, not available.

[a] Follow-up times (to recurrence or last contact) are reported as observed durations without censor-adjustment.

[b] Grade denotes Fuhrman or ISUP grading according to resection date.

Table S2. Composition and staining conditions of five multiplex immunofluorescence antibody panels.

Panel	Round	Fluorophore	Antibody	Dilution	Vendor (Cat. #)
**TME panel**	Round 1 (TSA)	TSA-488	Mouse anti-D2-40	1:100	Dako (M3619)
		TSA-555	Rabbit anti-PDGFRB	1:100	CST (3169)
		Alexa-647	Mouse anti-CD56	1:100	Dako (M7304)
		Alexa-750	Rabbit anti-CD11b	1:100	BioSB (6441)
	Round 2	Alexa-647	Mouse anti-CD20	1:100	Thermo (MS-340)
		Alexa-750	Rabbit anti-CD3	1:150	Thermo (MA5-14482)
	Round 3	Alexa-647	Rabbit anti-CD31	1:200	Abcam (ab28364)
		Alexa-750	Mouse anti-CD45	1:100	Dako (M0701)
	Round 4	Alexa-647	Pan-Epi cocktail:Rabbit anti-CAIX (CA9); Rabbit anti-E-Cadherin; Rabbit anti-panCK	1:200 each	NovusBio (NB100-417); CST (3195); Abcam (9377)
		Alexa-750	Mouse anti-HLA-DR	1:5 000	Abcam (ab20181)
**T cell panel**	Round 1 (TSA)	TSA-488	Mouse anti-PD-1	1:100	LSBio (B12784)
		TSA-555	Rabbit anti-CD3	1:750	Thermo (MA5-14482)
		Alexa-647	Rabbit anti-PD-L1	1:100	CST (13684)
		Alexa-750	Mouse anti-CD8	1:300	Dako (M7103)
	Round 2	Alexa-647	Rabbit anti-TIM-3	1:100	CST (45208)
		Alexa-750	Mouse anti-FOXP3	1:100	Abcam (ab20034)
	Round 3	Alexa-647	Rabbit anti-Granzyme B	1:200	Abcam (ab4059)
		Alexa-750	Mouse anti-Ki67	1:100	Dako (M7240)
	Round 4	Alexa-750	Pan-Epi cocktail:Rabbit anti-E-Cadherin; Rabbit anti-panCK	1:200 each	CST (3195); Abcam (9377)
**CAF panel 1**	Round 1 (TSA)	TSA-488	Rabbit anti-PDGFRB	1:100	CST (3169)
		TSA-555	Rabbit anti-PDGFRA	1:100	CST (5249)
		Alexa-647	Mouse anti-αSMA	1:200	Dako (M0851)
		Alexa-750	Rabbit anti-FAP	1:2 000	Abcam (ab207178)
	Round 2	Alexa-647	Pan-Epi cocktail:Rabbit anti-panCK; Rabbit anti-E-Cadherin	1:200 each	Abcam (9377); CST (3195)
**CAF panel 2**	Round 1 (TSA)	TSA-488	Goat anti-SPARC	1:500	R&D (AF941)
		TSA-555	Rabbit anti-PDGFRB	1:100	CST (3169)
		Alexa-647	Rabbit anti-Periostin	1:500	Abcam (ab215199)
		Alexa-750	Mouse anti-Vimentin	1:2 000	Dako (M0725)
	Round 3	Alexa-750	Pan-Epi cocktail:Mouse anti-panCK (Invitrogen MA5-13156); Mouse anti-panCK (BD 610182); Mouse anti-E-Cadherin (Abcam ab7753)	1:200 each	Invitrogen; BD; Abcam

Panel	Round	Fluorophore	Antibody	Dilution	Vendor (Cat. #)
**Macrophage panel**	Round 1 (TSA)	TSA-488	Rabbit anti-CD11c	1:2 000	Abcam (ab52632)
		TSA-555	Mouse anti-CD206	1:500	Proteintech (60143-1-Ig)
		Alexa-647	Rabbit anti-CD16	1:100	CellMarque (116R-14)
		Alexa-750	Mouse anti-CD68	1:100	CellMarque (168M-94)
	Round 2	Alexa-647	Mouse anti-CD45	1:100	Dako (M0701)
		Alexa-750	Rabbit anti-CD163	1:200	Abcam (ab188571)
	Round 3	Alexa-647	Mouse anti-HLA-DR	1:5 000	Abcam (ab20181)
	Round 4	Alexa-750	Pan-Epi cocktail:Rabbit anti-E-Cadherin; Rabbit anti-panCK	1:200 each	CST (3195); Abcam (9377)

TSA = Tyramide Signal Amplification

Table S3: Association of tumor epithelial FAP expression with epithelial marker intensity, immune and endothelial cell densities, and PD-L1 in localized ccRCC (n = 418)

Variable	FAP[a] neg (n=282)	FAP weak (n=89)	FAP strong (n=47)	p-value
**EpiStain (center)**				< 0.001
Low (n=135)	73 (27%)	35 (40%)	27 (59%)	
Med (n=135)	98 (36%)	27 (31%)	11 (24%)	
High (n=135)	102 (37%)	25 (29%)	8 (17%)	
**PD-L1[b] (tumor max)**				< 0.001
Neg (n=297)	230 (82%)	45 (51%)	20 (43%)	
Weak (n=98)	48 (17%)	36 (40%)	14 (39%)	
Strong (n=25)	4 (1%)	8 (9%)	13 (28%)	
**CD45[c] density**				< 0.001
Low (n=136)	110 (40%)	22 (25%)	4 (9%)	
Med (n=134)	97 (36%)	22 (25%)	15 (32%)	
High (n=135)	65 (24%)	43 (50%)	27 (59%)	
**CD31 density**				< 0.001
Low (n=137)	69 (25%)	38 (44%)	30 (65%)	
Med (n=135)	91 (34%)	33 (38%)	11 (24%)	
High (n=133)	112 (41%)	16 (18%)	5 (11%)	

[a]Tumor epithelial FAP maximum expression was scored per core (0 = negative; 1 = weak; 2 = strong) across two center and two border cores per patient; the highest core score was taken as the patient-level FAP score (n = 418).
[b]Tumor PD-L1 maximum expression was scored identically to FAP.
[c]$CD45^+$ and $CD31^+$ densities were calculated as fractions of epithelial-negative (Epi^-) cells in tumor-center cores (mean of two replicates), then categorized into tertiles (low/med/high) separately within the Helsinki and Turku cohorts before merging, yielding slight differences in n per category.
p-values by two-sided Pearson chi-square test

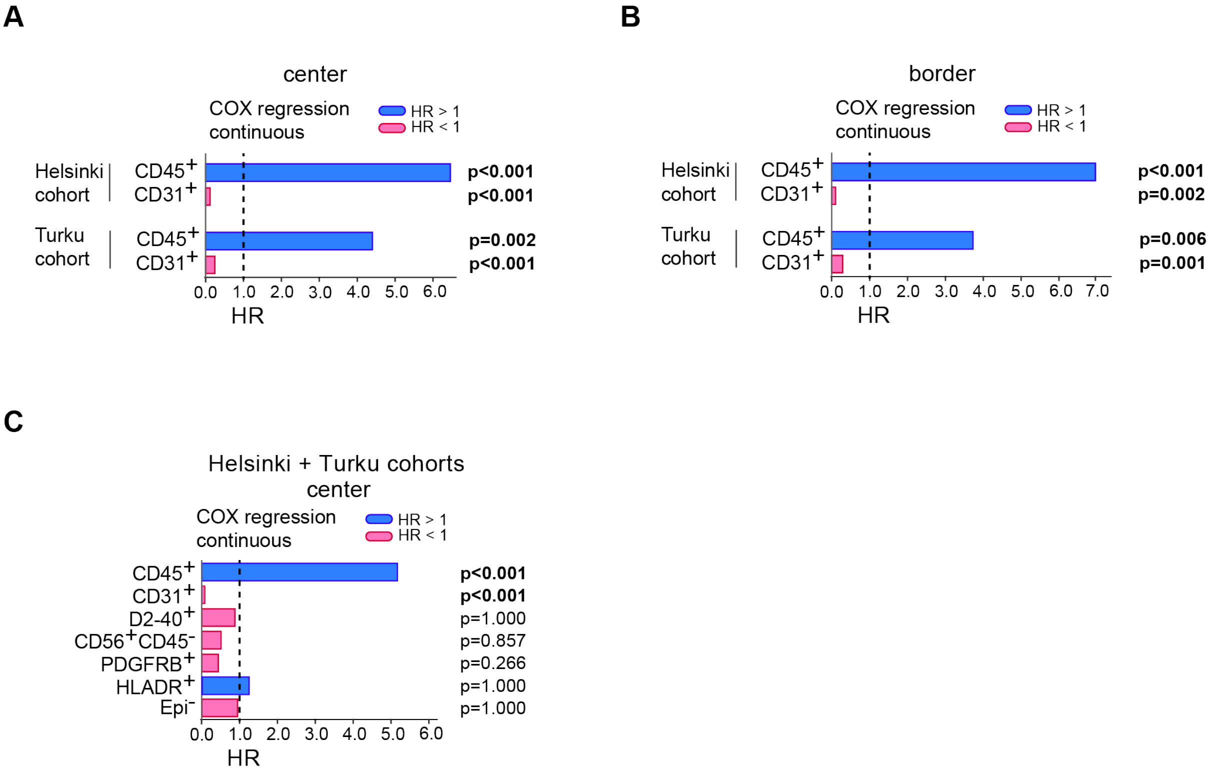

Figure S1. Univariate Cox regression of stromal and immune subset densities predicting recurrence-free survival in ccRCC.

(A) Univariate Cox proportional hazards analysis of continuous epithelial-negative (Epi^{-}) stromal and immune subset proportions in tumor-center cores from the Helsinki (n = 178) and Turku (n = 227) cohorts. Subset proportions are expressed as a fraction of Epi^{-} cells (non-epithelial). Hazard ratios (HR) with 95% confidence intervals (CI) are plotted on a logarithmic scale.

(B) Same analysis as in (A) for tumor-border cores (Helsinki n = 178; Turku n = 218).

(C) Univariate Cox analysis for the merged cohort of tumor-center cores (n = 405). Subset proportions are fractions of Epi^{-} cells, except the overall Epi^{-} fraction, which is expressed relative to total cells.

HR = hazard ratio; CI = confidence interval; p_cor = Bonferroni-corrected p-value; Epi^{-} = epithelial-negative.

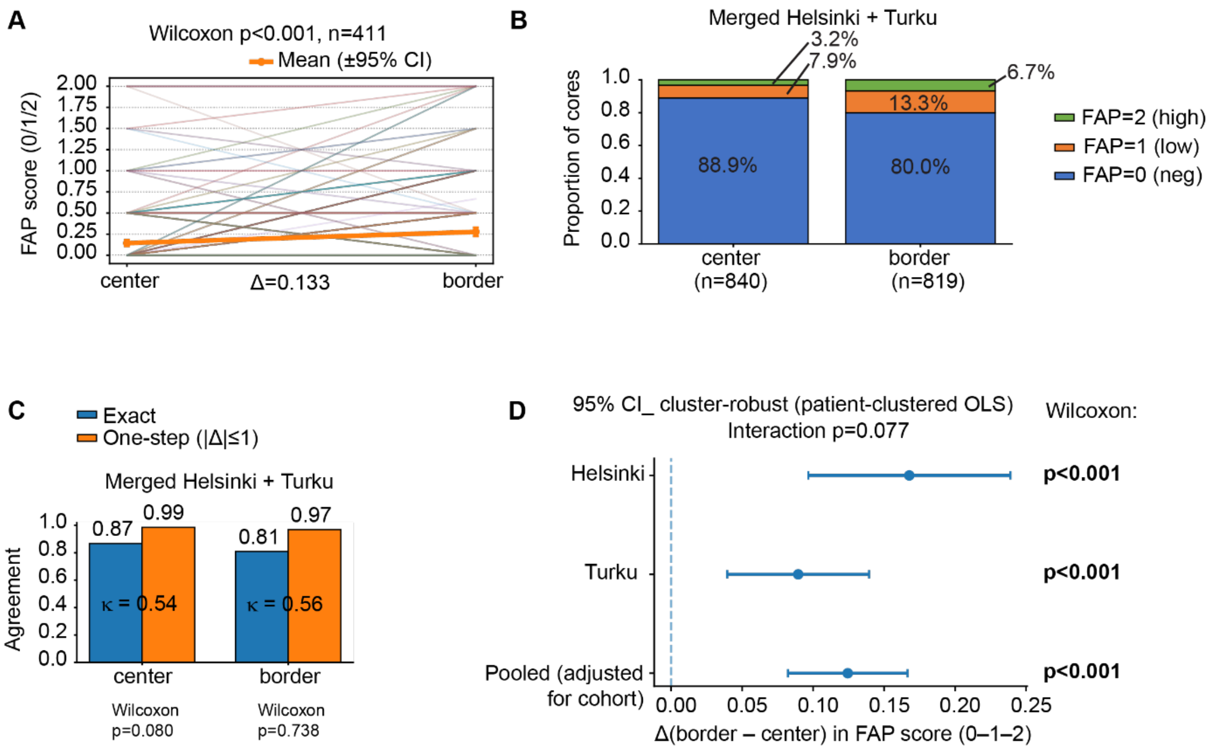

Figure S2. Spatial heterogeneity and within-region reliability of tumor-cell FAP.

(A) Within-patient center-to-border "spaghetti" plot of tumor-cell FAP (neg = 0/low = 1/high = 2; thick line/points show mean±95% CI (cluster-robust). Paired Wilcoxon on patient means: Δ(border–center) = 0.133, p = 1.8×10^{-9} (n = 411 patients; cores: center n = 840, border n = 819).

(B) Stacked proportions of FAP categories by region (FAP = 0/1/2), showing enrichment of higher categories at borders.

(C) Duplicate-core agreement within regions (center c1 vs. c2; border b1 vs. b2): exact and one-step ($|\Delta| \leq 1$) agreements and quadratic-weighted κ (center κ = 0.54; border κ = 0.56). No systematic shift within duplicates (Wilcoxon: center p = 0.080; border p = 0.738).

(D) Cohort-specific and pooled Δ(border–center) with patient-clustered robust 95% CIs; right-hand labels show cohort-wise p-values; "Interaction p" (subtitle) tests whether Δ differs between cohorts (here, not significant). Vertical dashed line marks Δ = 0. CI method: cluster-robust (patient-clustered OLS).

CI = confidence interval; OLS = ordinary least squares (with patient-clustered robust SEs where noted).

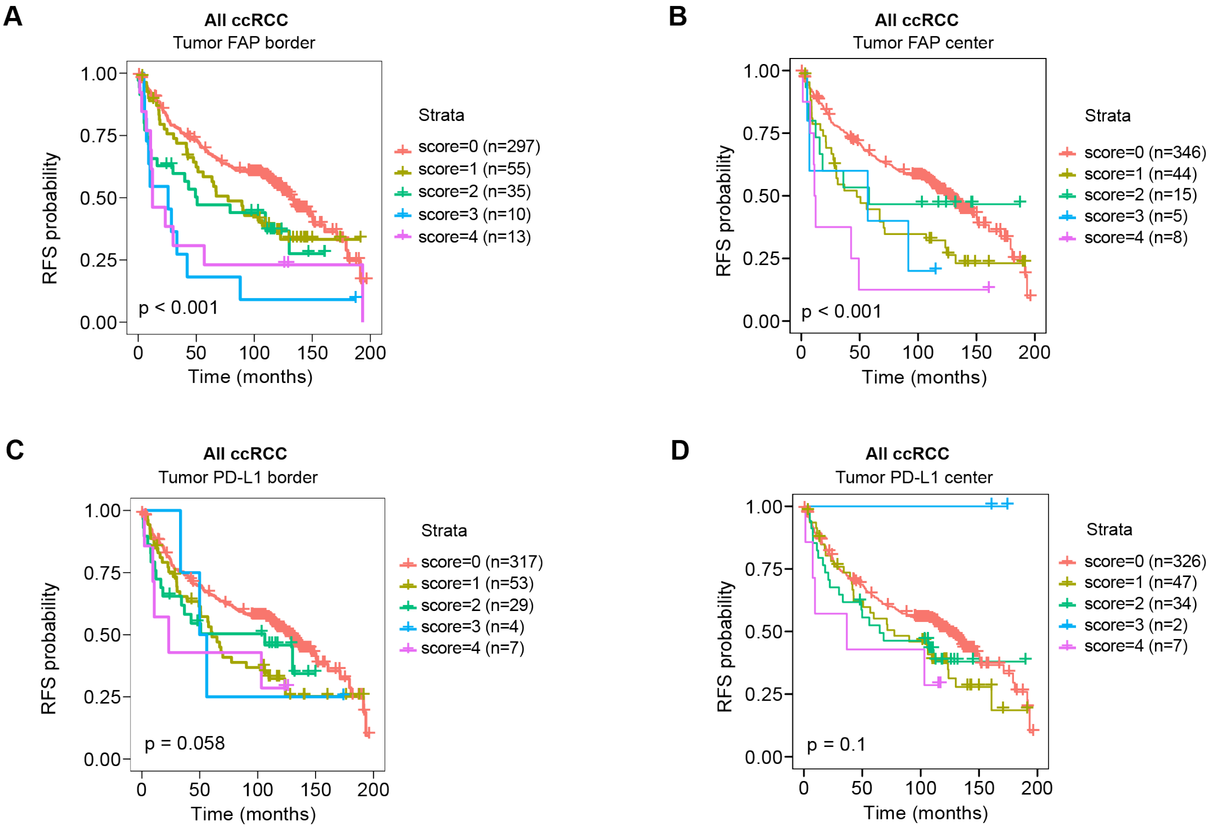

Figure S3. Tumor cell FAP but not PD-L1 expression stratify recurrence-free survival in localized ccRCC.

(A) Kaplan–Meier curves for recurrence-free survival (RFS) in all ccRCC patients (n = 410), stratified by cumulative tumor cell FAP score (0–4) determined from two tumor-border tissue microarray (TMA) cores per patient. Scores were obtained by summing immunostaining intensity (0 = negative, 1 = weak, 2 = strong) across replicate cores. Log-rank $p < 0.001$.

(B) Same analysis as in (A), using two tumor-center TMA cores per patient (n = 418). Log-rank p = 0.10 (not significant).

(C) Kaplan–Meier curves for RFS in all ccRCC patients (n = 410), stratified by cumulative tumor cell PD-L1 score (0–4) from two border cores. p = 0.058.

(D) Same analysis as in (C), using two center cores (n = 416). p = 0.42.

ccRCC = clear cell renal cell carcinoma; FAP = fibroblast activation protein; PD-L1 = programmed death-ligand 1; RFS = recurrence-free survival; TMA = tissue microarray.

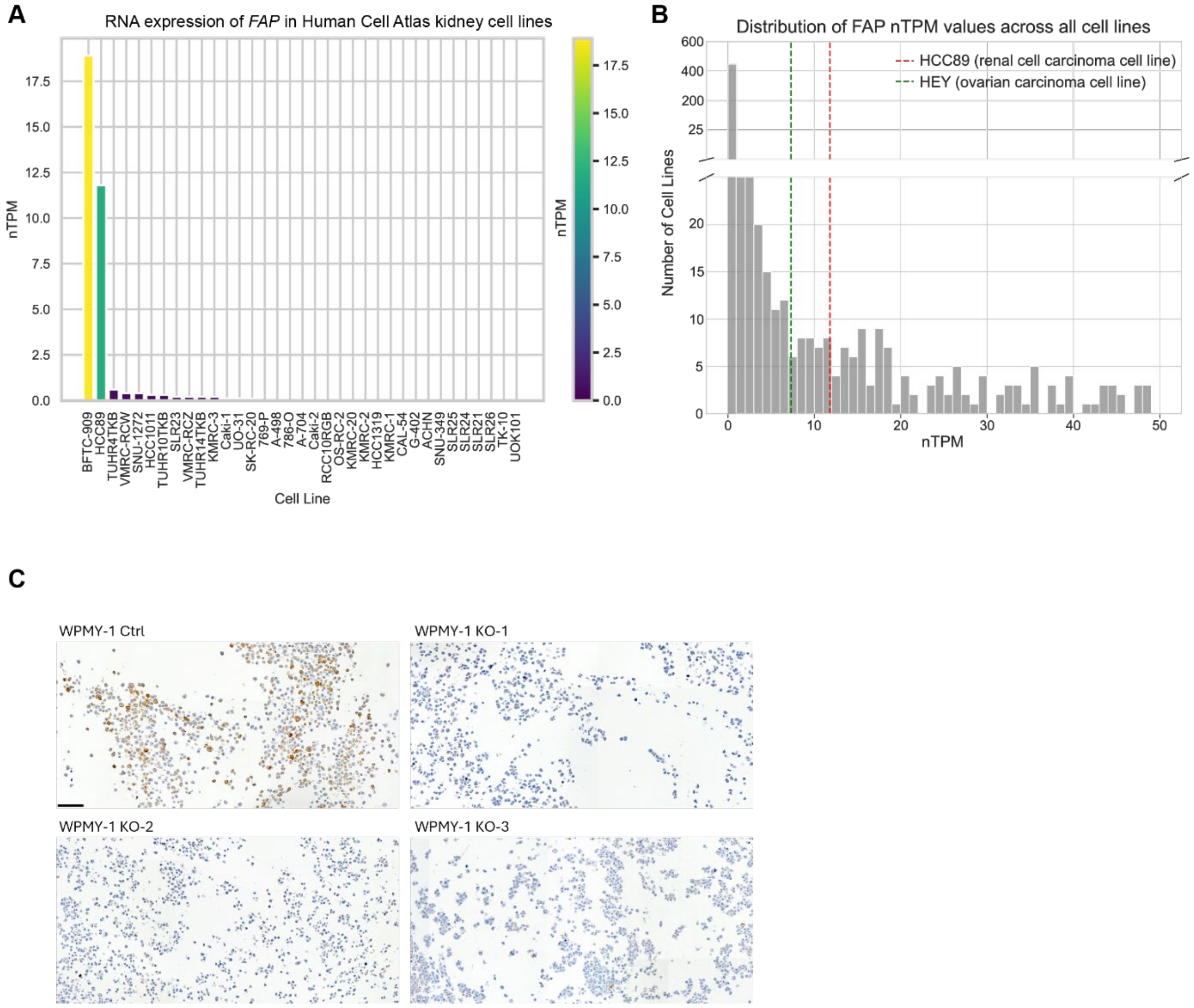

Figure S4. Validation of FAP antibody specificity and FAP expression profiling in cancer cell lines.

(A) FAP mRNA expression levels (normalized transcripts per million, nTPM) across kidney-derived cell lines from the Human Cell Atlas (The Human Protein Atlas v24.0). Data downloaded from https://www.proteinatlas.org/humanproteome/cell+line/data#cell_lines.

(B) Distribution of FAP nTPM values across all cell lines in the Atlas, with clear cell renal cell carcinoma line HCC89 (nTPM = 11) and ovarian carcinoma line HEY (nTPM = 7) highlighted as examples of high FAP expression [1].

(C) Immunohistochemical (IHC) validation of anti-FAP antibody (Abcam ab207178) on WPMY-1 fibroblast cells with CRISPR–Cas9 knockout of FAP. Wild-type control and three independent FAP KO clones were stained at 1:500 dilution. Scale bar = 100 µm.

IHC = immunohistochemistry; KO = knockout; nTPM = normalized transcripts per million; FAP = fibroblast activation protein.

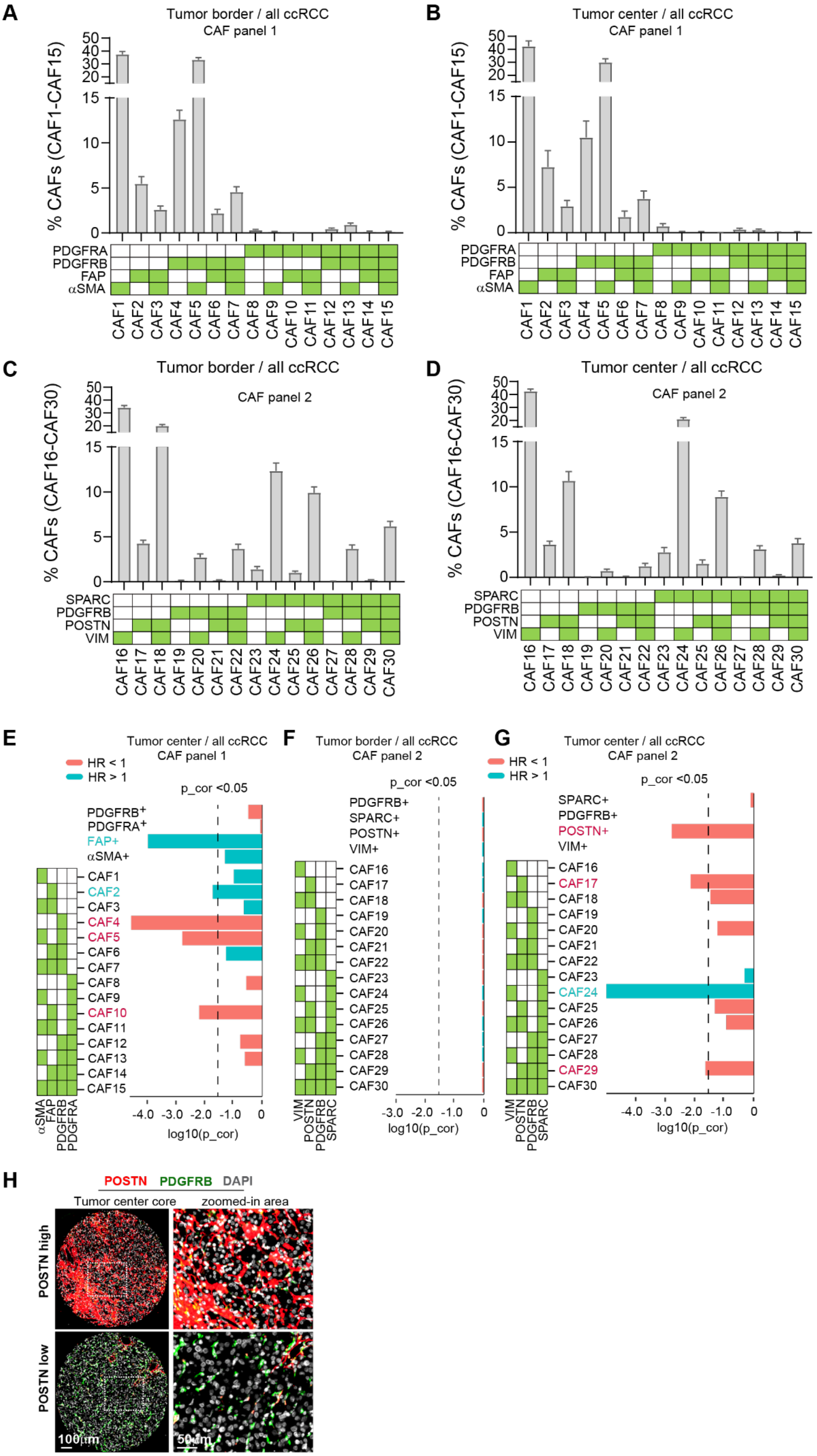
A
Tumor border / all ccRCC
CAF panel 1
% CAFs (CAF1-CAF15)
PDGFRA
PDGFRB
FAP
αSMA
B
Tumor center / all ccRCC
CAF panel 1
C
Tumor border / all ccRCC
CAF panel 2
% CAFs (CAF16-CAF30)
SPARC
PDGFRB
POSTN
VIM
D
Tumor center / all ccRCC
CAF panel 2
E
Tumor center / all ccRCC
CAF panel 1
HR < 1
HR > 1
p_cor <0.05
log10(p_cor)
F
Tumor border / all ccRCC
CAF panel 2
G
Tumor center / all ccRCC
CAF panel 2
H
POSTN PDGFRB DAPI
Tumor center core
zoomed-in area
POSTN high
POSTN low
100 μm
50 μm

Figure S5. Distribution and prognostic associations of multi-marker defined CAF subsets in localized ccRCC.

(A) Relative abundance of CAF panel 1 multi-marker subsets in tumor-border cores (mean of replicates; n = 394 ccRCC cases). Each subset (CAF1–CAF15) is expressed as a percentage of total CAFs; bars show mean ± 95% CI. Green shading denotes marker positivity defining each subset.

(B) Same as in (A), but for tumor-center cores (n = 414).

(C) Relative abundance of CAF panel 2 multi-marker subsets (CAF16–CAF30) in tumor-border cores (n = 408). Conventions as in (A).

(D) Same as in (C), but for tumor-center cores (n = 414).

(E) Univariate Cox proportional hazards regression for continuous proportions of CAF panel 1 subsets (all Epi^{-}) in tumor-center cores (n = 414), predicting recurrence-free survival (RFS). Points indicate hazard ratios (HR) on a $\log_2$ scale; horizontal lines are 95% confidence intervals (CI); p_cor is the Bonferroni-corrected p-value.

(F) Univariate Cox regression as in (E), but for CAF panel 2 subsets in tumor-border cores (n = 408).

(G) Univariate Cox regression for CAF panel 2 subsets in tumor-center cores (n = 414). Conventions as in E).

(H) Representative mIF images of POSTN high case (upper panels) and POSTN low case (lower panels) in tumor-center cores. Main panels scale bar = 100 µm; insets = 50 µm.

CAF = cancer-associated fibroblast; ccRCC = clear cell renal cell carcinoma; Epi^{-} = epithelial-negative; RFS = recurrence-free survival; HR = hazard ratio; CI = confidence interval; p_cor = Bonferroni-corrected p-value; mIF = multiplex immunofluorescence; POSTN = periostin; PDGFRB = platelet-derived growth factor receptor β.

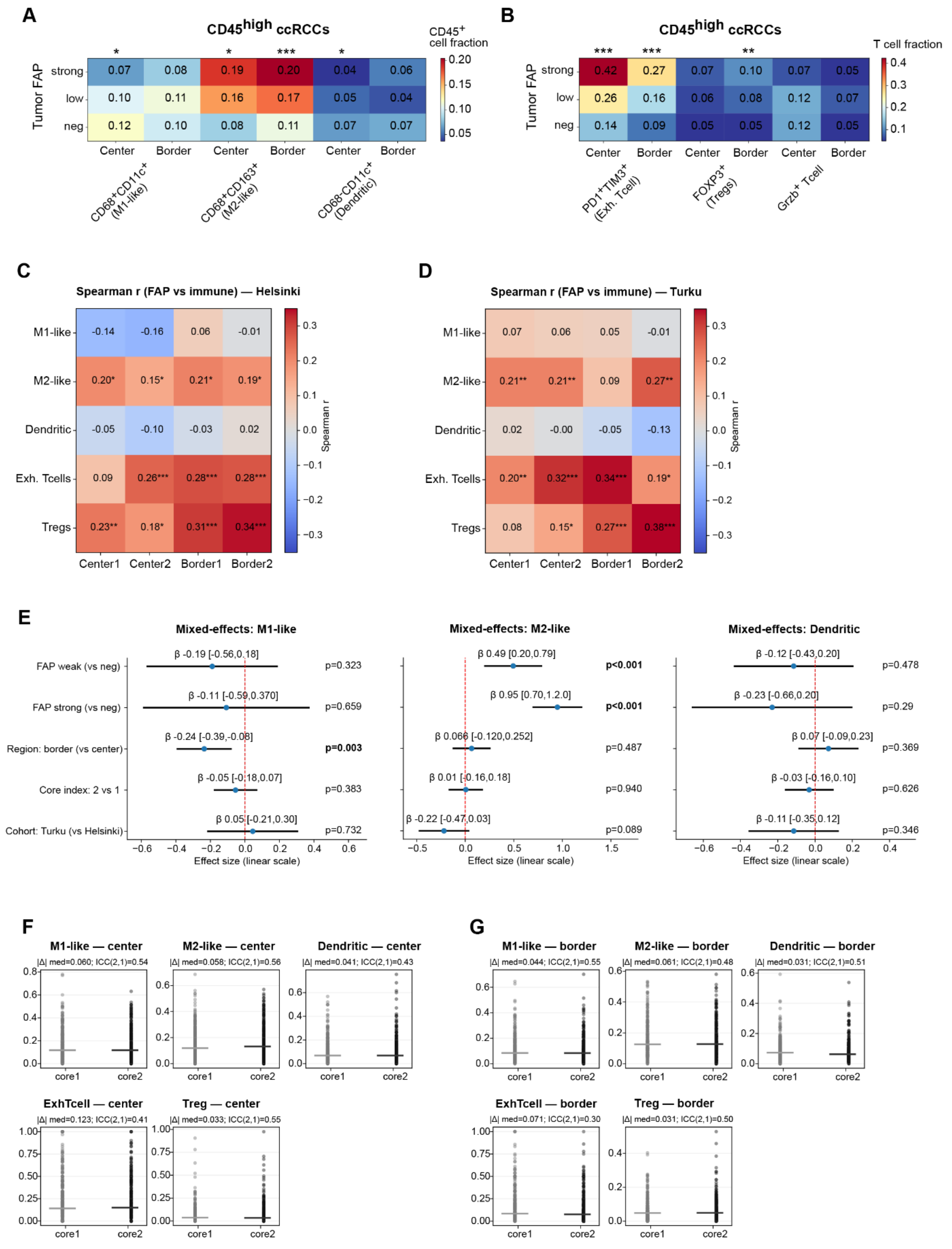

Figure S6. Cohort-specific correlations, mixed-effects for myeloid, and duplicate-core reliability. (A–B) Heatmap of myeloid subset fractions (Panel A) among CD45^{+} cells, and T-cell subset fractions (Panel B) among T cells stratified by tumor-epithelial FAP score (neg/weak/strong) in CD45-high ccRCC (center: n = 130–135; border: n = 124–133). *P < 0.05, **P < 0.01, ***P < 0.001; two-sided

Kruskal–Wallis.

(C–D) Per-core Spearman correlations between tumor-cell FAP score and immune fractions across the four cores. Heatmaps stratified by cohort (Panel C = Helsinki and Panel D = Turku). Cells show Spearman *r*; asterisks denote FDR-adjusted significance across cores per feature (Benjamini–Hochberg, 10% FDR). Helsinki per-core sample sizes: center n = 169–187; border n = 172–178. Turku per-core sample sizes: center n = 191–210; border n = 171–185.

(E) Mixed-effects Forest plots for myeloid fractions. Fixed effects: FAP (weak/strong vs. neg), region (border vs. center), core index (2 vs. 1), and cohort. Points show β (logit scale) with 95% CI; two-sided Wald *p* at right. Sample size n = 1456.

(F–G) Duplicate-core dispersion and reliability: core1 vs. core2 scatter/strip panels with median |Δ| and ICC(2,1) for center (left panel) and border (right panel). Sample sizes, Center: n = 320–365, ICC range 0.41–0.56; Border: n = 300–311, ICC range 0.30–0.55.

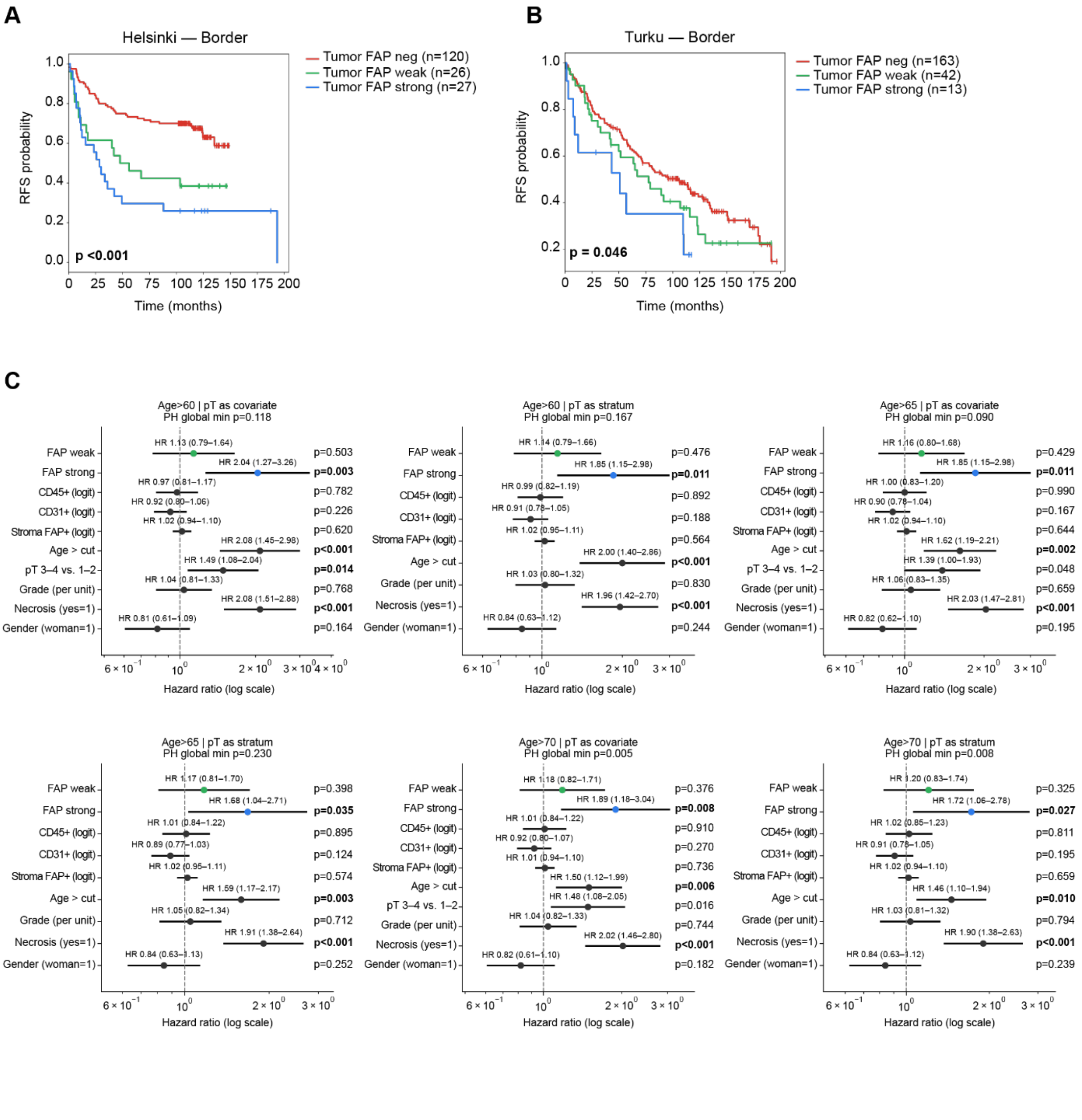

Figure S7. Supplemental survival analyses for tumor-cell FAP

(A–B) Kaplan–Meier curves for recurrence-free survival (RFS) by three-tier tumor-cell FAP score (negative, weak, strong) in tumor-border cores, shown separately for the Helsinki (A) and Turku (B) cohorts. Log-rank p-values are reported; group sizes (n) are indicated in the legends.

(C) Sensitivity of the cohort-stratified multivariable Cox model for RFS. Points depict hazard ratios (HRs) with 95% CIs for weak vs negative and strong vs negative tumor-cell FAP under alternative model specifications: age dichotomized at 60 / 65 / 70 years, and pT handled either as an ordinal covariate (1–4) or as a two-level stratum (1–2 vs 3–4). All models adjust for necrosis (yes/no), sex (woman = 1), grade (ordinal 1–4) and include stromal $CD31^{+}$ area, $CD45^{+}$ area, and stromal FAP% as continuous covariates (logit-scaled). The tumor-FAP effect (especially strong vs negative) remains directionally consistent and of comparable magnitude across specifications.

FAP, fibroblast activation protein; RFS, recurrence-free survival; HR, hazard ratio; CI, confidence interval; neg, negative.

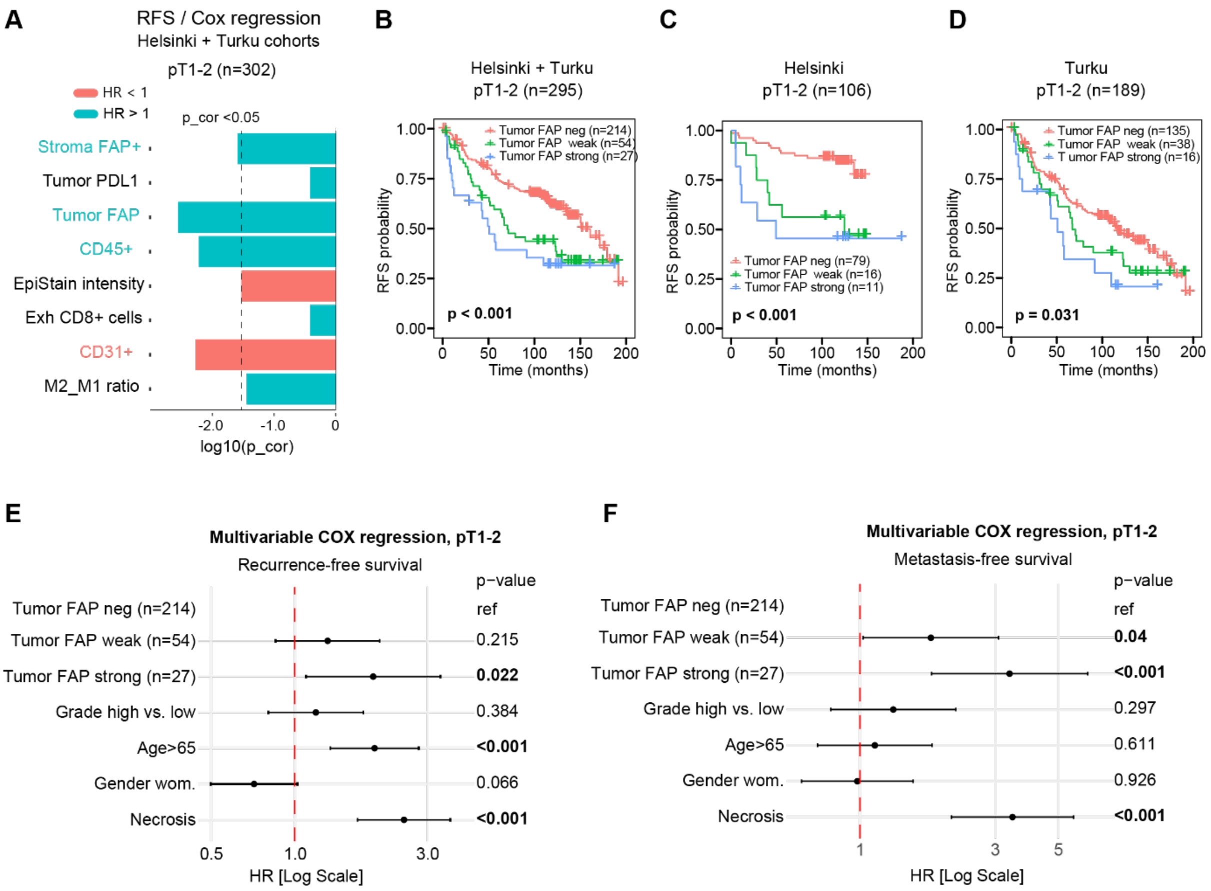

Figure S8. Tumor FAP independently predicts recurrence in early-stage ccRCC

(**A**) Box plot of univariate Cox proportional hazards regression for RFS in early-stage (pT1–2) ccRCC patients (n = 302) for selected variables. Bars extent to the left with HR) > 1 (increased risk) and for

HR < 1 (protective), with length equal to ($–\log_{10}$(p_cor)). Bars are colored cyan for HR > 1 and red for HR < 1. p_cor, Bonferroni-corrected p-value.
(**B-D**) Kaplan–Meier plots of RFS stratified by three-tier visual scoring of tumor cell FAP in early-stage ccRCC. Analysis for combined cohorts (B; n = 295)), Helsinki cohort (C; n = 106), and Turku cohort (D; n = 189). FAP scores determined as the maximum expression in either tumor center or border. p-values from log-rank test.
(**E**) Forest plot of multivariable Cox regression for RFS in early-stage patients (n = 302), including necrosis, sex, age > 65, Fuhrman grade, and tumor cell FAP (weak vs negative; strong vs negative).
(**F**) Forest plot of multivariable Cox regression for MFS in early-stage ccRCC (n = 302), using the same covariates. Conventions as in (E).

ccRCC = clear cell renal cell carcinoma; FAP = fibroblast activation protein; RFS = recurrence-free survival; MFS = metastasis-free survival; HR = hazard ratio; CI = confidence interval; p_cor = Bonferroni-corrected p-value; neg = negative.

REFERENCES

1. Li B, Ding Z, Calbay O, Li Y, Li T, Jin L, et al. FAP is critical for ovarian cancer cell survival by sustaining NF-kappaB activation through recruitment of PRKDC in lipid rafts. Cancer Gene Ther. 2023; 30: 608-21.
